# Fluorination strategy toward chemical and functional modification

**DOI:** 10.1016/j.fmre.2023.08.017

**Published:** 2023-12-20

**Authors:** Haotian Qiu, Shilie Pan, Miriding Mutailipu

**Affiliations:** a Research Center for Crystal Materials, Xinjiang Technical Institute of Physics and Chemistry, Chinese Academy of Sciences, Urumqi 830011, China; b Center of Materials Science and Optoelectronics Engineering, University of Chinese Academy of Sciences, Beijing 100049, China

**Keywords:** Fluorooxysalts, Fluorination, Fluorine, Nonlinear optical crystal, Inorganic synthesis

## Abstract

Fluorination is a powerful strategy for chemical and functional modification of materials because the introduction of fluorine atoms can alter the physical, chemical and electronic properties of a material and thereby result in improved key properties. The fluorination strategy has enabled the modification of material chemistry and properties at the microscale of fluorooxysalts, providing a diversity previously unattainable in oxysalts. Here, we review the recent progress, status, future opportunities, and challenges with concern of the chemical and material aspects for fluorooxysalts that contain fluorine-involved M-F bonds. The entire evolution of fluorooxysalts—from synthesis to structural chemistry and their functionality—is examined from the perspective of the polyanion. This review paper details how qualities of optical crystals, battery materials, and inorganic framework materials can be greatly enhanced by understanding the chemistry of inorganic fluorooxysalts. This review centers on the critical role that fluorine plays in the synthesis, characterization, and physical properties of these materials.

## Highlights

• In this review, the recent progress, status, future opportunities, and challenges with concern of the chemical and material aspects for fluorooxysalts that contain fluorine-involved M-F bonds are discussed.
• This review details how chemical insights into inorganic fluorooxysalts can broadly induce the property improvement for optical crystals, battery materials, and inorganic framework materials.
• The crucial role that fluorine plays in the synthesis, characterization, and physical properties of these materials is the core focus of this review.

## Graphical abstract

**Figure:**
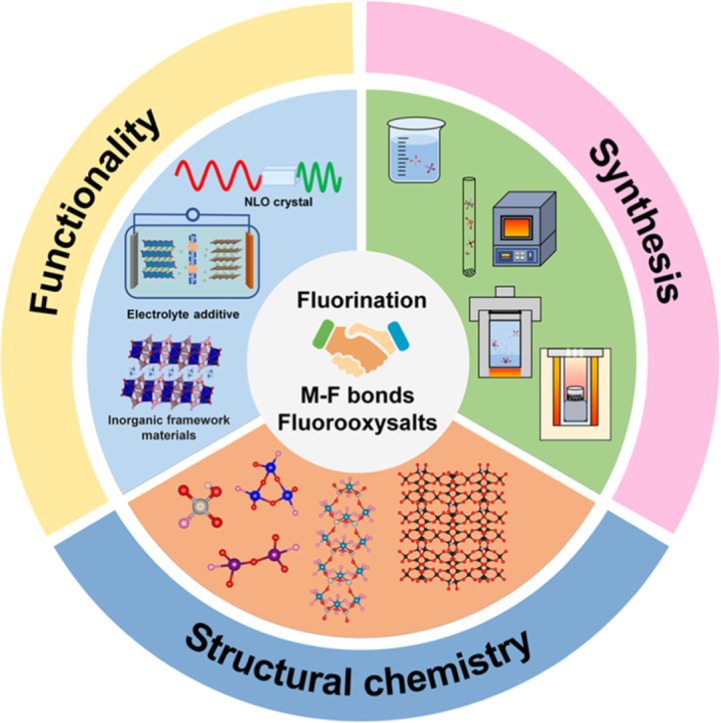

In this review, we summarize the recent progress, status, future opportunities, and challenges with concern of the chemical and material aspects of fluorooxysalts that contains fluorine-involved M-F bonds. This review details how chemical insights into inorganic fluorooxysalts can broadly induce the property improvement for optical crystals, battery materials, and inorganic framework materials.

## 1 Introduction

Inorganic oxysalts have long been the cornerstone of the development in chemistry and materials science, which are mostly composed of metallic cations and polyanions formed by nonmetallic ions and oxygens, including borates, carbonates, phosphates, nitrates, sulfates, silicates, iodates, arsenates, tellurates, and selenates [1], [2]. Because of their diverse crystal forms, inorganic oxysalts have a broad spectrum of chemical and physical characteristics that make them valuable in a variety of applications [3], [4], [5], [6]. Numerous technologies that make use of the special qualities of inorganic oxysalts have been developed as a result of the relationship that has been established between their crystal structure and properties. Researchers can adjust the chemical and physical characteristics of these materials for certain uses by adjusting the crystal structure [1], [2], [3], [4], [5], [6].

**Fig. 1:**
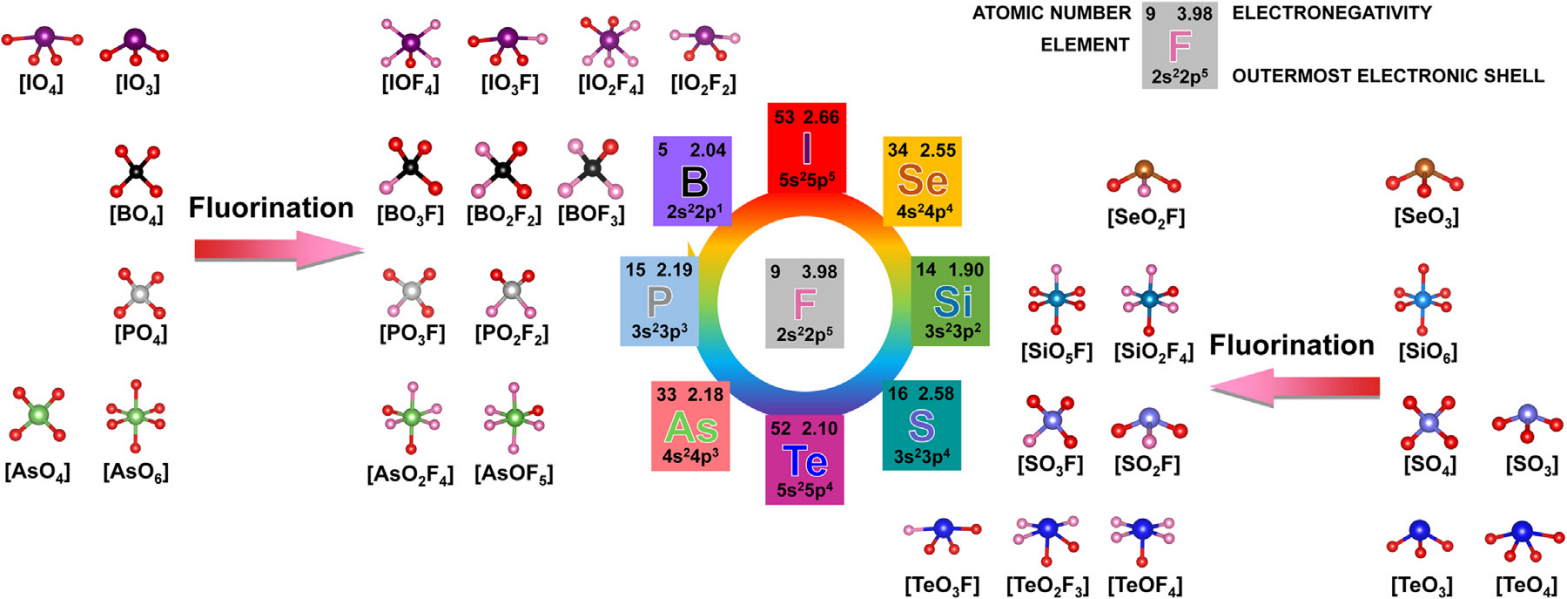
**The structure diagram of the formation of M-F bonds in fluorooxysalts.** In fluorooxysalts, a variety of fluorinated anionic units can be formed through partial substitution of oxygen atoms or by introducing new fluorine atoms into pure oxygen units. Since the elements of S, I, As, Te, and Se contain different valence states, the valence states in the fluorinated group here are S^6+^/S^4+^, I^5+^, As^5+^, Te^4+^ and Se^4+^.

Because of their functional changes, mixed anionic compounds with distinctive crystal structures have captured the interest of material scientists and chemists for the past century [7], [8], [9], [10], [11], [12]. The presence of several anions causes changes in the valence state, electronegativity, ionic radius, and coordination environment of the anions in mixed anion compounds. These modifications have a substantial impact on the materials' physicochemical characteristics and crystal structure, which makes them desirable for both industry and basic research [7], [8], [9], [10], [11], [12]. Oxyfluorides, oxynitrides, and oxyhydrides are examples of mixed anionic compounds that have demonstrated significant promise as functional materials in a variety of study areas, including energy storage, magnetism, optics, and more [7], [8], [9], [10], [11], [12]. The structural modification and performance optimization of the solid-state materials are popular in different functional materials, like effects of organic ligands and original mineral structures on properties of luminescent materials [13], [14]. Fluorine is known to have a strong electronegativity and small ionic radius, which could cause significant changes in bonding and crystal structure when incorporated into inorganic oxysalts. When fluorine is added to inorganic oxysalts, the coordination environment of metal or anionic polyhedra undergoes structural alterations that impact the materials' chemical and physical characteristics. In most cases, the fluorine atoms only replace certain oxygen atoms in the metal-oxygen polyhedra to form the metal-fluorine ionic bonds, resulting in the formation of oxysalt fluorides. In addition to natural minerals, synthetic oxysalt fluorides have been applied in various fields, including energy materials and optics [7], [8], [9], [10], [11], [12]. For example, borate fluoride KBe_2_BO_3_F_2_ is the only nonlinear optical crystal for deep-ultraviolet (deep-UV) laser output applications [15]. Phosphate fluoride A_2_FePO_4_F (A = Na, Li) served as a cathode in both Li-ion and Na-ion cells because of a large reversible capacity [16]. On the other hand, fluorine forms both metal-fluorine ionic bonds and nonmetal-fluorine covalent bonds when it partially substitutes the oxygen atoms on an anionic polyhedron in inorganic oxysalts. These substances, which go by the name fluorooxysalts, have special chemical and physical characteristics since they involve both kinds of bonding interactions [12]. In contrast to oxysalt fluorides, the fluorination of anionic groups in inorganic oxysalts modifies the original anionic framework and adds new primitives to the crystal structure (see Fig. 1). As a result, the materials' structural chemistry and physical characteristics will diversify, increasing their use in a range of industries including energy storage, sensors, and catalysis.

One could think of the conversion of inorganic oxysalts into fluorooxysalts as the outcome of a fluorination process. One effective method for changing a material's chemical and functional properties is fluorination [16], [17], [18], [19], [20], [21]. A material's electrical, chemical, and physical characteristics may change when fluorine atoms are added, leading to better performance than the original oxysalts. It has been demonstrated, for instance, that fluorine-doped spinels retain capacity better than oxide spinels at high cycle rates [17]. High reduction fluoride compounds can function as stabilizing agents throughout a broad voltage range, including those found in an electrochemical cell. This property has been incorporated into electrolyte design, both in salts (e.g., LiPF_6_) and additives (e.g., 4-fluoroethylene carbonate) within the electrolyte [18]. For electroactive materials, fluoro-organic lithium salts, such as (CF_3_SO_2_)_2_NLi and ((CF_3_)_2_(CHOSO_2_)_2_NLi, have been found to have attractive features as organic electrolytes in electroactive materials [19]. As cathode materials for sodium-ion batteries, fluorooxophosphates and fluorooxosulfates have been investigated; many of these compounds exhibit a minor unit cell expansion following intercalation. Because of their marginally higher insertion potential compared to oxygen-based phosphate and sulfate phases, sodium-ion batteries exhibit enhanced electrochemical performance and cycling stability [20]. When M-F bonds form in fluorooxophosphates and fluorooxosulfates, the symmetry is broken and the polarization anisotropy of stiff tetrahedral units is improved. This leads to materials with higher birefringence than their phosphate and sulfate counterparts in the field of optical materials [21]. Furthermore, the insertion of diverse [BOF] tetrahedra can control the original B-O anionic group arrangement to some degree in addition to leveraging the benefits of their microscopic optical features. Benefitting by this synergistic effect, fluorooxoborate has become an excellent candidate in the field of deep-UV functional crystals [22,23].

About 320 cases of fluorooxysalts (as shown in Table S1) have been recorded to date, which is a significantly less amount than the number of oxysalt fluorides. This implies that fluorooxysalt development is still in its infancy and that there is a great deal of room for future study in this field. Thus, now is the ideal moment to provide an overview of fluorooxysalts and explore how fluorine affects both chemical and functional modification. This paper describes how the chemical understanding of fluorooxysalts can be applied broadly to improve the properties of inorganic framework materials, optical crystals, and battery materials. To appreciate the impact of fluorooxysalts and their multiple connections to chemistry and materials science, it is useful to retrospect selected historical developments first, followed by a short introduction into synthesis before discussing structural chemistry and functionality.

## 2 Synthesis of fluorooxysalts

**Fig. 2:**
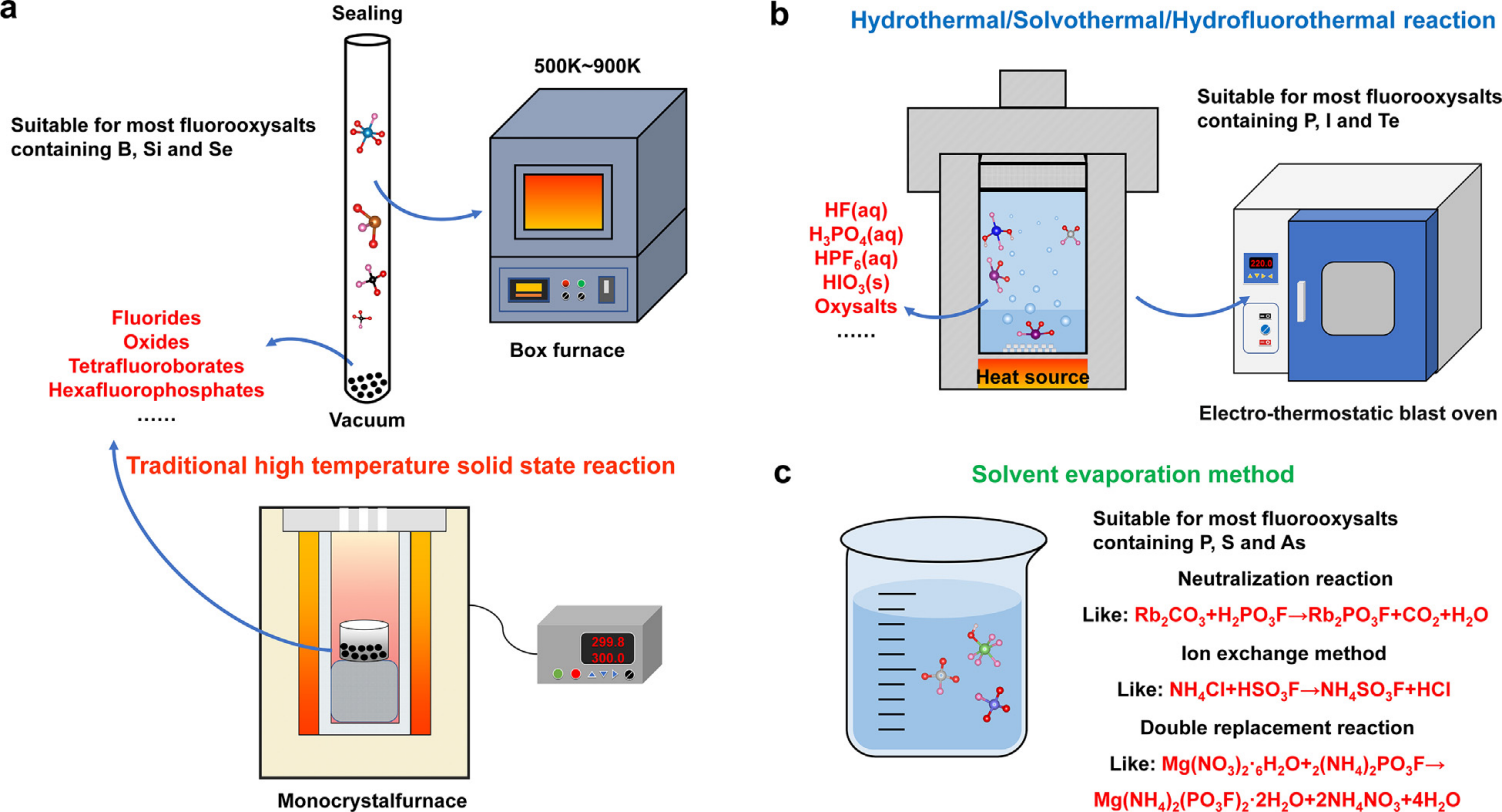
**Common synthetic methods for fluorooxysalts.** (a) Traditional high-temperature solid-state reactions. Owing to the volatile nature of fluorinated raw materials at high temperatures, a confined environment is usually necessary. Even if small amounts of fluorooxysalts can be formed by heating in open conditions, high reaction temperatures, such as above 900 K, need to be avoided. (b) Hydrothermal, solvothermal, and hydrofluorothermal reaction. The use of strong acid, high temperature, and high pressure sealed environment in hydrothermal reactors can quickly dissolve insoluble substances, such as alkaline earth metals and some transition metal fluorides and carbonates. (c) Solvent evaporation method. The solubility of the raw material in water, the pH of the solution, and the rate of evaporation of the solvent usually need to be considered, and for some fluorinated raw materials, glass containers should be avoided as far as possible.

Fluorooxysalts can be synthesized in three primary ways, similar to oxysalt synthesis: solvent evaporation method, mild hydrothermal reaction, and traditional high temperature solid state reaction (Fig. 2). The intended crystal structure and the final products' physicochemical characteristics can influence the synthesis of fluorooxysalts. It is crucial to remember that there are no hard and fast rules when it comes to selecting a synthesis method for a certain fluorooxysalt, even though different synthesis techniques may be employed for distinct elements. However, the majority of them go as follows: (i) due to their high melting points and thermal stability, high-temperature solid-state reactions are frequently used to create fluorooxysalts containing boron, silicon, and selenium; (ii) solvent evaporation or sol-gel methods are frequently used to create fluorooxysalts containing phosphorus, sulfur, and arsenic, though other synthesis techniques, like hydrothermal reactions, may also be employed; (iii) hydrothermal reactions are typically used to create fluorooxysalts containing phosphorus, iodine, and tellurium; however, other synthesis techniques may also be employed depending on the particular compound being created. Ultimately, the particular required qualities and traits of the finished goods determine which synthesis process is used. The synthesis of fluorooxosilicates and fluorooxoborates can be difficult due to the high melting point of borates and silicates since these compounds have labile properties at high temperatures that are comparable to those of tetrafluoroborates and hexafluorosilicates. During the synthesis process, a closed reaction system is usually needed to stop fluorine from leaking. Furthermore, in order to avoid undesirable side effects or the compound's breakdown, a lower reaction temperature might be required. To guarantee the stability and purity of the finished product, these parameters have to be carefully taken into account while synthesizing fluorooxosilicates and fluorooxoborates (Fig. 2a). In 2009, Jansen's group dried and mixed B_2_O_3_ and LiF into a gold crucible and sealed them in a glass ampoule in an argon atmosphere. The container was then heated to 673 K, kept warm, and gradually brought down to the room temperature. With this, the first lithium fluorooxoborate LiB_6_O_9_F was successfully synthesized [24]. Analogously, from 1994 to 2003, they obtained single crystals of three fluoroselenites and three anhydrous fluorotellurates by this similar method, raw materials of which also included non-metallic oxides and metallic fluoride [25], [26], [27], [28]. Subsequently, researchers have adopted a similar way to synthesize a series of fluorooxoborates in recent years [29], [30], [31], [32], [33], [34], [35], [36], [37], [38].

The number of known alkaline-earth fluorooxoborates with high melting points is smaller than that of known alkali metal fluorooxoborates with lower melting points due to the restrictions of high reaction temperatures in the synthesis of metal fluorooxoborates. This implies that because of the difficulties in synthesizing them, there might be a large number of unidentified fluorooxoborates that have not yet been investigated. Metal fluorooxoborates are usually synthesized by high-temperature solid-state processes, but some alkali metal fluorooxoborates, such as A_10_B_13_O_15_F_19_ (A = K and Rb) and CsB_4_O_6_F [34,37], have been generated via the melt growth approach in open systems at lower temperatures. To grow single crystals of these compound, a combination of ingredients is melted and then slowly cooled. But not all fluorooxoborates can be treated with this technique; in particular, those with high melting points or high vapor pressure at high temperatures could not work well with it.

Hydrothermal and solvothermal reactions are important branches of inorganic synthetic chemistry, and have become important means of powder preparation, synthesis of new compounds, and crystal growth [39,40]. Due to its low reaction temperature and strong reaction activity, the subcritical hydrothermal reaction (about 100–300°C) has been widely employed in the synthesis of fluorooxoiodates and fluorooxophosphates to date (Fig. 2b). Using the hydrofluorothermal approach, Weller's group produced a series of transition metal monofluorooxophosphates between 2011 and 2013. The major raw ingredients employed in this process were metal fluoride, hexafluorooxophosphate, and a few organic solvents [41,42]. Furthermore, a case of hydroxyfluorooxoarsenate GaF(AsO_2_F(OH))_2_ was synthesized by means of a comparable technique in 2015. In the production of fluorooxoiodates, such as SrI_2_O_5_F_2_, Ba(IO_2_F_2_)_2_, and A_2_MoO_2_F_3_(IO_2_F_2_) (A = Rb and Cs) [43,44], HIO_3_ and H_5_IO_6_ are frequently utilized as iodine sources and hydrofluoric acid as fluorine sources. Similar to this, some hydroxyfluorooxotellutates, such as HgTeO_2_F(OH) and BaF_2_TeF_2_(OH)_2_, can be produced by a hydrothermal reaction employing TeO_2_ as a tellurium source and HF as a fluorine source [45,46].

The evaporative solvent method is comparatively easy and effective when compared to the other two methods. Its reaction mechanism primarily consists of the double decomposition, ion exchange, and neutralization reactions. Certain fluorooxophosphates can also be produced by solvent evaporation in addition to hydrothermal synthesis (Fig. 2c). For example, Chen et al. reported the hydrothermal synthesis of ammonium monofluorooxophosphate (NH_4_)PO_3_F, which was subsequently utilized as a precursor to react with various bases and salts to create a variety of alkali metal, alkaline earth metal, and complex monofluorooxophosphates [47]. It is simple to synthesize different monofluorooxophosphates with distinct crystal structures and characteristics by using (NH_4_)PO_3_F as a precursor. Compared to conventional solid-state reactions, this technique has a number of benefits, including easier purification of the finished product and better control over the reaction parameters. Usually, fluorosulfonic acid and metal halides, such as sulfates or chlorides, react to produce fluorooxosulfates. Some metal fluorooxosulfates are formed when the halide ion is replaced by a fluorosulfate ion as a result of this reaction. Using this technique, Thompson et al. synthesized a few fluorooxosulfates of alkali and alkaline earth metals as early as 1964, and they later published the crystal structures associated with those compounds [48]. Reports on the hydrothermal reaction-based synthesis of fluorooxosulfates have surfaced recently [49]. This strategy might present fresh opportunities for investigating and creating unique fluorooxosulfate molecules. In the study of solid-state chemistry, a material's structure is a major factor in defining its characteristics and its uses. After reviewing the pertinent synthesis techniques, the rich structural chemistry of fluorooxysalts will be covered in detail.

## 3 Structural chemistry of fluorooxysalts

**Fig. 3:**
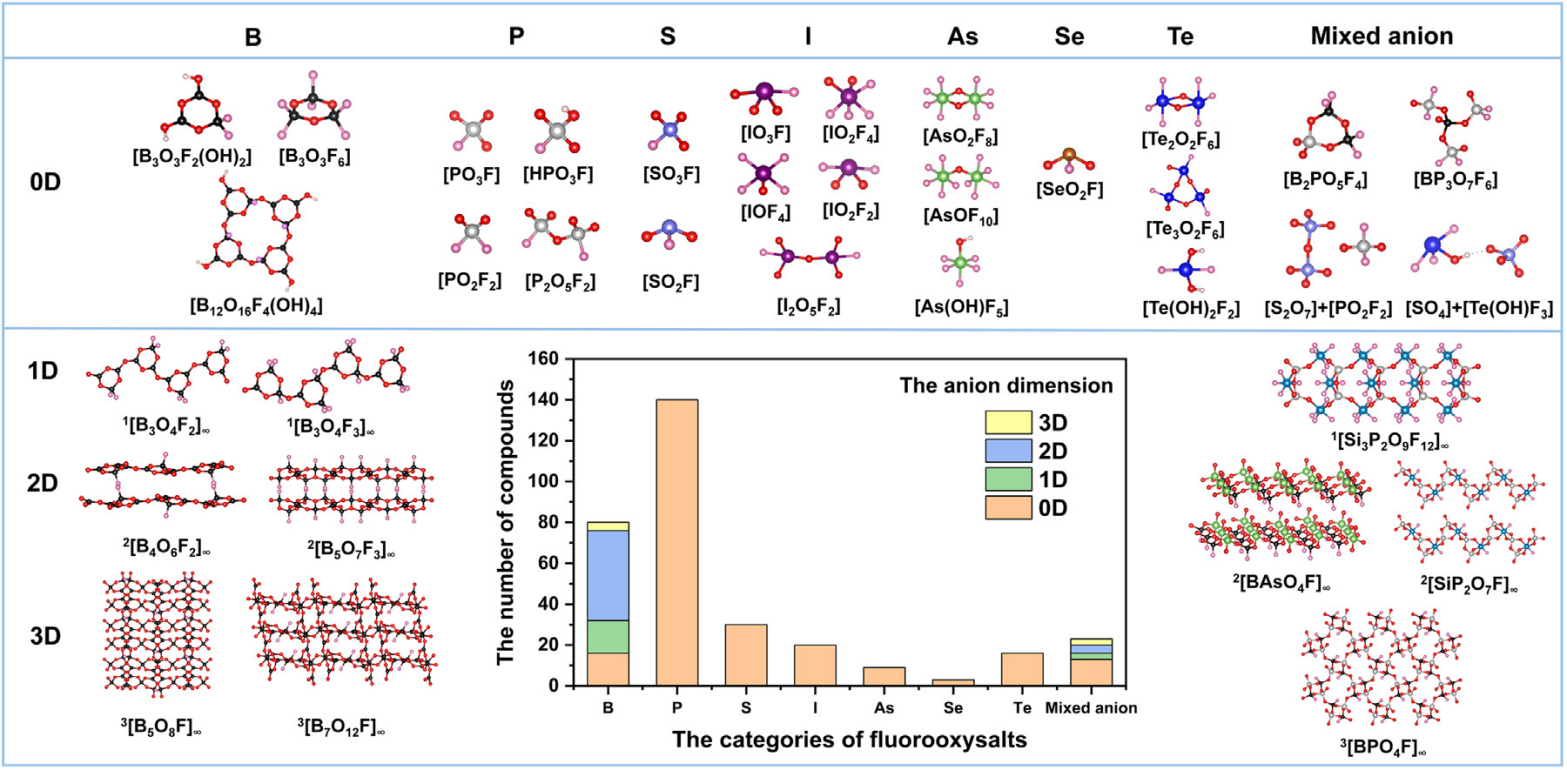
**Structural chemistry of fluorooxysalts with different dimensionality of anionic frameworks.** The statistical numbers of fluorooxysalts in different anionic dimensionality are from the ICSD and COD databases. All of the structure diagrams were drawn by using the corresponding crystallographic information file that stored in the ICSD.

According to the different fluorinated anion groups, fluorooxysalts can be divided into fluorooxoborates, fluorooxophosphates, fluorooxosulfates, fluorooxosulfites, fluorooxoiodates, fluorooxoarsenates, fluorooxotellurates, fluorooxoselenites, and mixed anionic fluorooxysalts. Among the known fluorooxysalts, more than 80% contain fluorinated covalent [MO_4-n_F_n_] (n = 1-3) tetrahedra, where M refers to central atoms such as B, P and S (Fig. 3). It is well known that borates and phosphates with rigid tetrahedra ([BO_4_] and [PO_4_]) tend to form polymeric anion framework under the effect of bridging oxygens [1]. The incapacity of fluorine atoms to coordinate with two or more central atoms limits the number of bridging oxygens when the fluorinated tetrahedra are added to the systems mentioned above, hence decreasing the anionic framework's dimensionality. For example, the anionic framework of fluorooxophosphates and fluorooxosulfates usually consists of isolated fluorinated tetrahedra ([PO_3_F], [PO_2_F_2_], and [SO_3_F]) or dimers ([P_2_O_5_F_2_]) [50], [51], [52], some of which also contains OH^−^ (in [HPO_3_F]) [53]. For many years, sodium monofluorooxophosphate Na_2_PO_3_F has been used as an ingredient in toothpaste and as a corrosion inhibitor in concrete reinforcement [54,55]. In Na_2_PO_3_F, the isolated [PO_3_F] tetrahedra fill the pores of a three-dimensional (3D) network formed by various Na-based polyhedra, including [NaO_6_] octahedra, [NaO_5_F] and [NaO_6_F] polyhedra, through edge-sharing or face-sharing. The detailed crystal structure of fluorooxosulfates (KSO_3_F) was first reported by Trotter et al., in 1967 [56]. Similarly, the isolated [SO_3_F] tetrahedra fill the pores of a 3D network formed by the [KO_8_F_2_] polyhedra through edge-sharing connection model.

Unlike phosphates and sulfates, which typically only contain tetrahedral units, borates can contain various structural units including [BO_2_] linear units, [BO_3_] planar units, and [BO_4_] tetrahedra [1]. As a result, borates have a wider variety of structural characteristics than phosphates and sulfates. The [BO_3_] and [BO_4_] units in borates can form varied anionic frameworks from 0D to 3D by sharing vertices or edges. With the introduction of fluorinated [BO_4-n_F_n_] (n = 1-3) tetrahedra, fluorooxoborates show rich structural chemistry, with 0D to 3D anion frameworks accounting for 22%, 19%, 53%, and 6%, respectively. Obviously, the proportion of fluorooxoborates with 3D anionic framework is very small, which verifies the shear action of fluorinated tetrahedra on the B-O units, thus reducing the dimensionality of anionic framework. Fluorooxoborate Na_3_B_3_O_3_F_6_ has been reported since the 1950s [57], but its crystal structure was not studied until 2012 by Jansen et al. [58]. The cyclo-fluorooxotriborate fundamental building unit (FBB) of Na_3_B_3_O_3_F_6_, [B_3_O_3_F_6_], is constructed by three [BO_2_F_2_] tetrahedra linked together in a cyclic arrangement. Interestingly, Na_3_B_3_O_3_F_6_ is the first fluorooxoborate with a 0D anion framework, meaning that its anionic units are not connected in 1D, 2D, and 3D networks, respectively. Additionally, it is the first known fluorooxoborate to have an anionic framework formed entirely by the [BOF] fluorinated tetrahedra. About half of the known fluorooxoborates have 2D layered anionic frameworks, which typically extend along a crystal plane through covertex connections between [BO_3_], [BO_4_], and [BO_4-n_F_n_] units. Examples of such compounds include AB_4_O_6_F (A = NH_4_, Na, Rb, Cs) and MB_5_O_7_F_3_ (M = Mg, Ca, Sr) [29,[31], [32], [33], [34], [35],38].

Fluorooxoborates have various anionic frameworks, but they can also take on different crystal structures or polymorphs due to pleomorphism. This is because tunable crystal formations are made possible by the impact of fluorinated tetrahedra on the structure's microscopic symmetry. BaBOF_3_ fluorooxoborate polymorph is such a case reported by our group [59]. All of them possess the same FBB of a [BO_2_F_2_] tetrahedron and secondary building block (SBB) of a [FBa_4_] tetrahedron, whereas [FBa_4_] SBBs can form 2D ^2^[Ba_2_F_2_]_∞_ wavy layers. Moreover, both of them consist of 1D ^1^[BOF_2_]_∞_ infinite chains and ^2^[Ba_2_F_2_]_∞_ wavy layers, and the 1D infinite ^1^[BOF_2_]_∞_ chains and ^2^[Ba_2_F_2_]_∞_ layers arrange alternately to generate the 3D structure of these compounds. Through the analysis, the structural changes of three phases come from the different orientation and arrangement of the 1D ^1^[BOF_2_]_∞_ infinite chains in their lattice.

Besides the common fluorinated tetrahedral units, there are other fluorinated polyhedra in fluorooxysalts. As early as 1940, Rogers et al. discovered a case of fluorooxoiodate KIO_2_F_2_ [60], whose anionic framework is composed of isolated [IO_2_F_2_] fluorinated units with the shape of a triangular bicone. Up to now, fluorooxoiodates have been found to contain four different types of fluorinated units: [IO_2_F_2_] [60], [IO_3_F] [61], [IOF_4_] [62], and [IO_2_F_4_] [63]. These fluorinated units exist in isolation to form a 0D anion framework [43,[60], [61], [62], [63]. It is remarkable that all fluorooxoiodates crystallize in the orthomorphic or monoclinic system, and the proportion of non-centrosymmetric space group was as high as 40%. In 1980, a metal-free fluorooxoiodate was synthesized by Slim et al. [64], which consists of two [IOF_2_] and two [IO_2_F_4_] anions linked by asymmetric oxygen bridges to give a cyclic molecule. Tellurate and iodate have similar structures because their elements are adjacent to each other in the periodic table. When pure oxygen primitives [TeO_3_] and [TeO_4_] are fluorinated, three kinds of fluorinated units can be formed, *i.e*, [TeO_3_F], [26] [TeO_2_F_3_], [27] and [TeOF_4_] [65]. Although there are only 10 cases of fluorooxotellurates with 0D anionic framework that are stored in the ICSD, which reflects diverse structural chemistry. In anhydrous fluorooxotellurates, KTeOF_3_ contains two phases, that is, non-centrosymmetric *P*2_1_ phase and centrosymmetric *P*4_2_/*n* phase. In the structure of *P*4_2_/*n* phase, [TeO_2_F_3_] units form a dimer [Te_2_O_2_F_6_] through a common edge, while [TeO_2_F_3_] exists in isolation in the *P*2_1_ phase structure. For hydroxyfluorooxotellurates, [Te(OH)F_4_] [66], [TeO_2_F(OH)] [46], and [TeF_2_(OH)_2_] [45] units have been found to date. While there are no reports on the synthesis or properties of fluoroselenates, there are examples of fluoroselenite compounds with intermediate oxidation states. For example, only 3 cases of fluorooxoiselenites (ASeO_2_F (A = K, Rb and Cs)) were included in ICSD, all of which were reported by Jansen et al. in 1994 [25]. The triangular conical [SeO_2_F] unit is isolated in the metal-oxygen polyhedra's interstitial region in their architectures. Due to the fact that sulfur and selenium are near on the periodic table, sulfite systems, such as fluorooxosulfite, also contain structures that are comparable to selenite. They have a triangular cone [SO_2_F] in their structures that resembles fluorooxoselenite.

Earlier, compounds with either oxyfluoride [SiO_x_F_4−x_] (x = 1, 2, 3) tetrahedra or [SiO_x_F_6−x_] (x = 1, 2, 3, 4, 5) octahedra have only been reported in fluorinated organic compounds. Until 2018, our group successfully synthesized the first fluorooxosilicophosphate K_4_Si_3_P_2_O_7_F_12_, which consists of unprecedented *cis*-[SiO_2_F_4_] species [67]. Structurally, K_4_Si_3_P_2_O_7_F_12_ exhibits the unique 1D ^1^[Si_3_P_2_O_9_F_12_]_∞_ anionic chains along the [100] direction, which afford two types of rings, that is, [SiP_2_O_7_F_4_] composed of a dipolyphosphate [P_2_O_7_] group and a [Si(1)O_2_F_4_] octahedral group, and [Si_2_P_4_O_14_F_8_] composed of two dipolyphosphate [P_2_O_7_] groups and two octahedral [Si(2)O_2_F_4_] groups. Subsequently, *trans*-[SiO_2_F_4_] and [SiOF_5_] units were discovered in another fluorooxosilicophosphates Na_4_Si_2_PO_4_F_9_ and CsSiP_2_O_7_F by Luo's group [68,69]. In addition, arsenate, similar to silicate, also contains fluorinated units, *i.e*, [AsO_2_F_4_] [70] and [AsOF_5_] [71].

It is important to note that mixed anionic fluorooxysalts are fluorooxosilicophosphate compounds that contain two or more anionic units, including fluorooxoborophosphate ([BOF]+[BO_3_]/[BO_4_]+[PO_4_]), [72,^73^,[79], [80], [81] fluorooxophosphorborate ([POF]+[BO_3_]/[BO_4_]), [74] fluorooxoboroarsenate ([BOF]+[AsO_4_]), [75] sulfate-fluorooxophosphate ([POF]+[SO_4_]), [76] fluoroborate-fluorooxophosphate ([BF_4_]+[POF]), [77] and sulfate-fluorooxotellurate ([SO_4_]+[TeOF]) [78]. The majority of mixed anionic fluorooxysalts (∼60%), whose anionic frameworks are made up of [BOF] and [PO_4_] units, are fluorooxoborophosphates, of which there have only been roughly 20 examples identified. In 2004, the first case of inorganic fluorooxoborophosphates NH_4_[BPO_4_F] was reported by Zhao et al. [79]. In the anionic framework of NH_4_[BPO_4_F], [BO_3_F] and [PO_4_] units share corners and form helical ribbons of ^1^[BPO_4_F]_∞_ along the 2_1_ screw axes of the *P*2_1_3 space group. Subsequently, Jansen et al. obtained two Na_3_B_2_PO_5_F phases *via* Lewis acid-base reaction, and the appearance of this polycrystalline phenomenon reflects the rich structural diversity of mixed anionic compounds [80]. Two phases crystallize in the monoclinic *P*2_1_/*n* and orthogonal *Cmcm* space groups, respectively, which features the same cyclic [B_2_PO_5_F_4_] polymers constructed by [BO_2_F_2_] and [PO_4_] units. The difference is that the polymers are near parallel in **II** phase, whereas the rings have large dihedral angles in **I** phase. Whereafter, a series of fluorooxoborophosphates containing only tetrahedral units were synthesized, such as AB(PO_4_)F (A = K, Rb and Cs) [72,73]. In 2022, our group introduced the π-conjugated [BO_3_] units into this system for the first time, obtaining a new fluorooxoborophosphate (NH_4_)_3_B_11_PO_19_F_3_ with a 2D layered structure [81]. The FBB of (NH_4_)_3_B_11_PO_19_F_3_, that is, [B_5_PO_14_F] unit, is polymerized to unprecedented ^2^[B_5_PO_10_F]_∞_ layers. Up to now, fluorooxoborophosphate compounds' anionic dimensionality has been extended from 0D to 2D in recent years, highlighting the intricate structural chemistry of these substances. In addition, three new clades, namely, fluorooxophosphorborates ([POF]+[BO_3_]/[BO_4_]), fluorooxophosphorsulfates ([POF]+[SO_4_]), and fluoroborate-fluorooxophosphate ([BF_4_]+[PO_3_F]), were found in the mixed anionic fluorooxysalts [74,^76^,77]. Each branch, however, only occurs once, and from the appropriate tetrahedral units, they all form 0D anion frameworks. We are curious about the qualities that fluorooxysalts, with their rich structural chemistry, will have in a solid form. In the sections that follow, we'll concentrate on these qualities and talk about how fluorine affects them.

## 4 Optical properties of fluorooxysalts

### 4.1 Second harmonic generation

**Fig. 4:**
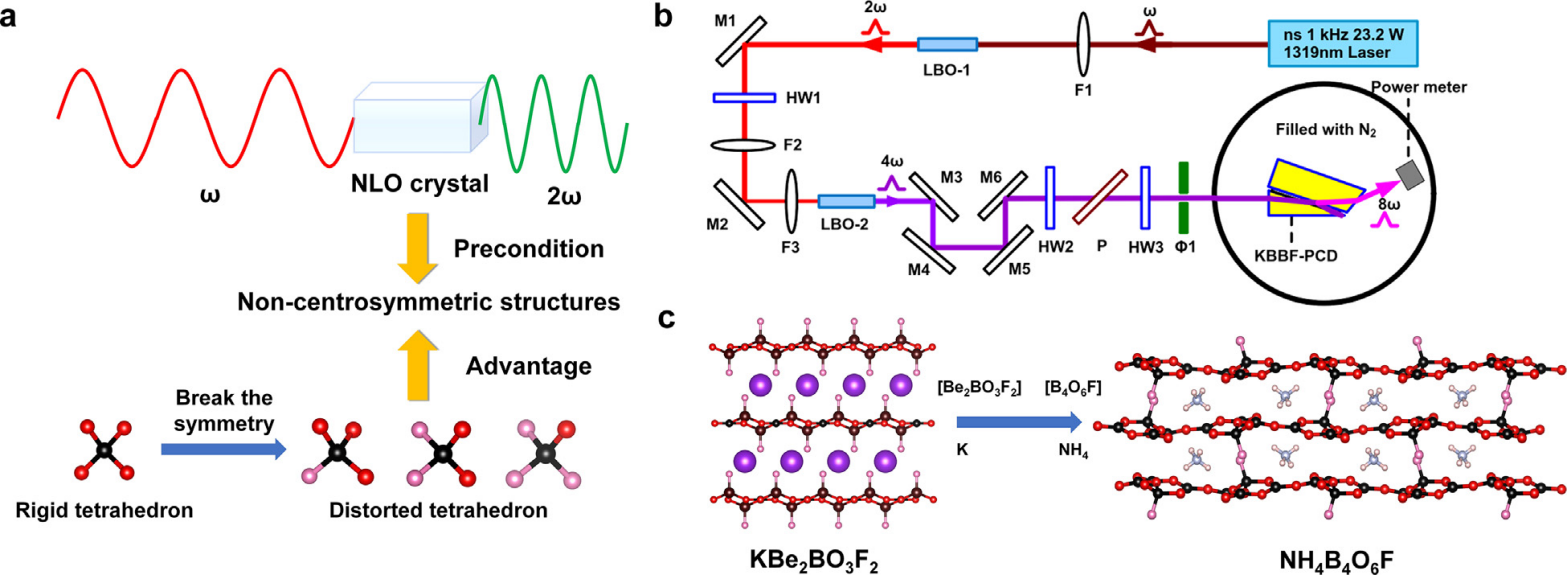
(a) The fluorinated tetrahedra in the anions are more favorable for producing a non-centrosymmetric structure. (b) Light path diagram for the generation of 2.14 mW deep-ultraviolet laser at 165 nm by eighth-harmonic generation of a 1319 nm Nd: YAG laser using KBBF crystal. Reproduced with permission from ref. [86]. Copyright 2016 IOP Publishing. (c) Structural evolution of KBBF to ABF.

Since the advent of nonlinear optics, which serves as the foundation for all solid-state lasers and the carrier of second harmonic generation (SHG), nonlinear optical crystals have found extensive application in a variety of industries, including photochemical synthesis, biomedicine, and laser lithography [82], [83], [84]. A non-centrosymmetric space group is required for the crystallization of nonlinear optical crystals in order to produce the second order nonlinear optical effect [82], [83], [84]. Because they contain fluorine atoms, fluorinated tetrahedra have been shown to break their own symmetries more readily than pure oxygen tetrahedra. This is due to the fact that fluorine is smaller and more electronegative than oxygen, which causes a larger distortion of the tetrahedral structure and, consequently, non-centrosymmetric formations [21], [22], [23]. The lack of chirality in fluorinated tetrahedra can lead to a high fraction of centrosymmetric structures even though these units can violate their own symmetry and form non-centrosymmetric structures. Many different structural configurations are available when groups surrounding the fluorinated tetrahedra are arranged inconsistently. Some of these configurations may even show centrosymmetric characteristics. This is especially true for compounds like fluorooxophosphates and fluorooxosulfates, which have fluorinated tetrahedra present but a significant fraction of centrosymmetric structures (16% and 20%). Conversely, up to 44% of fluorooxoborates have fluorinated tetrahedra in their non-centrosymmetric proportion. Centrosymmetric structures are obviously not produced in fluorooxoborates FBBs synthesized by numerous anionic groups. It is similarly simple to create non-centrosymmetric structures from polyhedra with substantial distortion when considering non-tetrahedral fluorinated units (Fig. 4a). Currently, roughly 40% of fluorooxoiodates have non-centrosymmetric structures with four distinct fluorinated units. The study of new nonlinear optical crystals is aided by the generally high chance of non-centrosymmetric structures produced by fluorooxysalts.

One crucial crystal parameter that must be considered in order to increase laser conversion efficiency is the second order nonlinear optical coefficient. $\chi_{ij}$ [83], [84], [85]. According to the Chen's anionic group theory, the size of $\chi_{ij}$ depends on the geometric superposition of the microscopic anionic groups [85]. It has been established in earlier research that π-conjugated units ([BO_4_], [PO_4_], [SO_4_], and [SiO_4_]) contribute more to the SHG response than non-π conjugated rigid tetrahedral units ([BO_3_], [CO_3_], and [NO_3_]). Chen et al. studied the microscopic second-order polarizability of common anionic groups in borates, where the value of [BO_3_] is much higher than that of [BO_4_] units. Among available borates, KBBF family (Fig. 4b) is the only crystal that can output deep-UV coherent light through a sixfold frequency [86]. The excellent linear and nonlinear optical properties of KBBF stem from the 2D ^2^[Be_2_BO_3_F_2_]_∞_ layers composed of [BO_3_] and [BeO_3_F] units. In 2017, our group discovered a promising candidate of deep-UV crystal NH_4_B_4_O_6_F (ABF) in fluorooxoborates [29]. Structurally, ABF can be seen as a derivative of KBBF, *i.e.*, the 2D ^2^[Be_2_BO_3_F_2_]_∞_ layers and K^+^ cations are replaced by the 2D ^2^[B_4_O_6_F]_∞_ layers composed of [BO_3_] and [BO_3_F] units and NH_4_^+^ cations, respectively (Fig. 4c). In terms of property, the SHG coefficient of ABF is remarkably larger than that of KBBF (about × 2.5 KBBF), meanwhile, ABF and KBBF have similar shortest phase matching wavelengths which affects the wavelength range of the laser output from the crystal.

The [BO_3_] groups in ABF are the main source of the large SHG response, according to first-principles calculations; the microscopic optical characteristics of the [BO_3_F] units have also drawn interest. Simultaneously, the optical properties of three fluorinated units [BO_4-n_F_n_] (n = 1-3) were calculated based on the fluorooxoborates reported previously [34], [35], [36], [37], [38], [39], [40], [41], [42], [43]. It is worth noting that the hyperpolarizability in connection with the SHG response of the three fluorinated groups is much higher than that of the [BO_4_] group or even exceeds than that in [BO_3_] groups. In order to verify the positive effect of fluorinated groups on the SHG response, a series of new non-centrosymmetric fluorooxoborates were synthesized afterwards, including NaB_4_O_6_F, [33] RbB_4_O_6_F, [31] CsB_4_O_6_F, [34] SrB_5_O_7_F_3_, [35] CsKB_8_O_12_F_2_, [31] CsRbB_8_O_12_F_2_ and MB_2_O_3_F_2_ (M = Sn, Pb) [31,36]. The examination of the true contribution of [BOF] groups to the SHG effect is impacted by the fact that the majority of fluorooxoborates contain [BO_3_] linked to [BOF] units. Although MB_2_O_3_F_2_ (M = Pb, Sn) exhibits a remarkably strong SHG response and their anionic framework contain only the uniformly arranged [BO_3_F] units, the enhancement of SHG effects is mainly attributed to the active 6*s*^2^ lone-pair electrons. It is noteworthy that PbB_2_O_3_F_2_ has the largest SHG effect in fluorooxysalts (13 × KDP), originating from the distortion directions of the NLO-active charge densities for Pb^2+^ cations and [BO_3_F] tetrahedra are along the *c*-axis. Of course, efforts in theoretical prediction also indicate that [BOF] can be used as NLO-active units, [87] for example, an artificial SrB_2_O_3_F_2_ consisting exclusively of tetrahedral [BO_3_F] units has a very large SHG effect of about 3.4 × KDP with the band gap of > 8.0 eV. Nevertheless, similar to other covalent [MO_4_]/[MO_3_F] (M = Li, Al, Be, Zn) tetrahedra, [88] the presence of [BO_3_F] facilitates the modulation of the coplanar orientation of the [BO_3_] groups in a uniform arrangement, resulting in the formation of layered anion frameworks in quantity.

Other fluorooxysalt systems, such as fluorooxophosphates, fluorooxoiodates, fluorooxotellurate, fluorooxooxosilicophosphates, and fluorooxoborophosphates, have progressively drawn the interest of chemists and materials scientists due to the application of fluorination strategy in the exploration of new nonlinear optical crystals. There aren't enough non-centrosymmetric inorganic fluorooxosulfate samples available to investigate their nonlinear optical characteristics. In 2018, our group and Wu's group analyzed the microscopic optical properties of anionic groups [PO_4-m_F_m_] (m = 1-2) in fluorooxophosphates, and the results show that compared with the rigid pure oxidized [PO_4_] groups, the optical properties of the two fluorinated groups were improved [89], [90], [91]. Relevant experiments confirm this, remarkably strong SHG intensities of (NH_4_)_2_PO_3_F, [C(NH_2_)_3_]_2_PO_3_F, and NaNH_4_PO_3_F·H_2_O were measured to be 0.9, 1.0, and 1.1 × KDP under 1064 nm and approximately 0.2, 0.2, and 0.3 × BBO under 532 nm, respectively [89].

When compared with the nonlinear optical crystals applied in the UV regions, like borates and phosphates, metal iodates exhibit a much wider transparent range (up to 12 μm), reaching the middle infrared spectral region. Therefore, iodates have the potential as a visible-mid-infrared nonlinear optical crystal. In 2016, Qin et al. reported the two metal iodates RbIO_3_ and RbIO_2_F_2_, which exhibit SHG effects of about 20 and 4 times that of KDP, respectively [92]. The arrangement of the [IO_3_] and [IO_2_F_2_] accounts for their notable differences. The parallelism of fluorinated [IO_2_F_2_] units is much less than that of [IO_3_] in RbIO_3_, resulting in a much smaller net dipolarity. In order to study the nonlinear optical properties of fluorooxoiodates in detail, our group calculated the microscopic optical properties of four fluorinated groups. Compared with [IO_3_] units, [IO_2_F_2_] and [IO_3_F] show comparable and larger hyperpolarizability, respectively, while the other two primitives contain smaller hyperpolarizability. Therefore, subsequent reports on fluorooxoiodates also confirm the superiority of the first two groups, such as [GaF(H_2_O)][IO_3_F] (10 × KDP, 4.34 eV) and A_2_MoO_2_F_3_(IO_2_F_2_) (A = Rb/Cs) (5.0/4.5 × KDP, 3.77/3.43 eV) [93,94]. Like fluorooxoiodate, fluorooxotellurate with distorted polyhedra is also a potential system for nonlinear optical crystals. In 2020, a UV nonlinear optical fluorooxotellurate crystal BaF_2_TeF_2_(OH)_2_ designed by the band-gap engineering was reported by Halasyamani et al. [45]. The physical property measurements show that this crystal could effectively balance the requirements among the short UV absorption edge (∼205 nm), large SHG response (∼3 × KDP), and moderate birefringence (∼0.078@350-700 nm). Compared with many tellurates, BaF_2_TeF_2_(OH)_2_ possesses a wider range of light transmittance (0.205-8.5 μm). In addition, the optical properties of mixed anionic fluorooxysalts have also been studied. CsSiP_2_O_7_F is the sole nonlinear optical active fluorooxosilicophosphate with deep-UV transmission, with the structure consisting of an unprecedented [SiP_2_O_10_F] moiety containing hexacoordinate [SiO_5_F] species [69]. In the anionic framework, [SiP_2_O_10_F] moieties are linked to each other via −Si–O–P– linkages to form a corrugated ^2^[SiP_2_O_7_F]_∞_ layer. The SHG response of CsSiP_2_O_7_F is approximately 0.7 × KDP, which is close to thoseo of other deep-UV transparent NLO silicates. According to theoretical calculations, the contribution to SHG effect mainly comes from [SiP_2_O_10_F] units; however, the contribution of [SiO_5_F] primitives to the SHG effect was not discussed in detail. The study on the nonlinear optical properties of fluorooxoborophosphates began with AB(PO_4_)F (A = NH_4_, K, Rb and Cs) series compounds, [72,73] they display SHG signals that are approximately 0.3, 1, 0.4 and 0.6 × KDP, respectively. Although their anionic groups are [BO_3_F] and [PO_4_], KBPO_4_F is not isomorphic to other three compounds. In KBPO_4_F, each downward [BO_3_F] tetrahedron is surrounded by three upward [PO_4_] tetrahedra by sharing common O-atom corners, and further forming a 2D layered structure along (001) plane. In other three compounds, they are isomorphic, so their structures are similar to that of NH_4_BPO_4_F mentioned earlier. It is the preferred arrangement of tetrahedra in KBPO_4_F that leads to the larger SHG effect. (NH_4_)_3_B_11_PO_19_F_3_ is the first deep-UV NLO fluorooxoborophosphate, which exhibits a large SHG effect (1.2 × KDP) and short phase matching wavelength (∼190 nm) [81]. In (NH_4_)_3_B_11_PO_19_F_3_, the presence of [BO_3_F] eliminates the dangling bonds of [BO_3_] and regulates its arrangement, leading to the blue shift of UV cutoff edge and the increase of SHG response.

### 4.2 Birefringence

An anisotropic crystal will normally split a light beam into two polarized components, one of whose vibration directions is perpendicular to the other. We refer to this phenomenon as double refraction or birefringence. The birefringence phenomena can now be used to achieve two main goals: modifying polarized light and achieving angle phase matching to fulfill the needs of nonlinear optical crystals at various optical wavelengths [95,96]. Birefringent crystals for use in UV, visible, and infrared wavelengths have grown more sophisticated during the last few decades [1]. The necessity for birefringent crystals in this band is increasing with the advent of deep-UV lasers. In order to meet the requirements of their intended applications, deep-UV birefringent crystals are often designed and synthesized with at least two of the following properties: (i) sufficiently large birefringence; (ii) adequately short UV cutoff edge (corresponding to wide bandgap) and high transmittance for the operating wavelength.

Greater optical anisotropy and birefringence are favoured by π-conjugated units having larger polarizability anisotropy than non-π conjugated units, according to prior theoretical simulations of the microscopic optical characteristics in different anionic groups [88]. Thus, as we investigate new deep-UV birefringent crystals, oxysalts with π-conjugated units—that is, borates, carbonates, and nitrates—have drawn more and more attention. However, because dangling bonds are present, oxysalts that only contain isolated π-conjugated units might have little to no transmission in the deep-UV spectral region. The addition of hydroxyl or covalent tetrahedra to the terminal oxygen atoms has shown to be a successful tactic in avoiding this issue. As mentioned earlier, many fluorooxoborates have π-conjugated [BO_3_] and covalent [BOF] tetrahedra, which favor the formation of layered structures parallel to each other to produce large optical anisotropy in the crystals, [1,[29], [30], [31], [32], [33], [34], [35] such as BaB_8_O_12_F_2_ (*Δn*_cal_*_._* = 0.116@1064 nm, λ_cutoff_ < 180 nm) [97]. In recent years, the appearance of hydroxyfluorooxoborates provides a new way to explore new deep-UV birefringent crystals. The introduction of multiple hydroxylated π-conjugated units ([BO_2_(OH)] and [B(OH)_3_]) based on fluorooxoborates not only benefits the elimination of dangling bonds and the increase of polarizability anisotropy, but also provides hydrogen bond donors, which may be helpful in regulating crystal structure [98,99]. In 2021, the first sodium difluorodihydroxytriborate-boric acid Na[B_3_O_3_F_2_(OH)_2_]·[B(OH)_3_] was reported by our group [98]. Structurally, two isolated groups [B_3_O_3_F_2_(OH)_2_] and [B(OH)_3_] are connected by hydrogen bonds and arranged parallel to the [011] direction, where the π-conjugated units generate a large in-plane anisotropy, resulting in a large birefringence (*Δn_cal._* = 0.1252 at 546 nm). However, the lower symmetry of this crystal (No. 2 *P*$\bar{1}$) limits the processing and application of crystals. Subsequently, another case of hydroxyfluorooxoborates (NH_4_)_4_[B_12_O_16_F_4_(OH)_4_] as a potential deep-UV birefringent crystal was also reported by our group [99]. Different from Na[B_3_O_3_F_2_(OH)_2_]·[B(OH)_3_], (NH_4_)_4_[B_12_O_16_F_4_(OH)_4_] crystallizes in a uniaxial crystal system with a tetragonal space group *P*4/*ncc* (No. 130) so that birefringence could be measured by polarizing microscopy method (*Δn*_obv._ = 0.12@546.1 nm). Structurally, the [B_3_O_4_F(OH)] moiety, formed by three anionic groups ([BO_3_], [BO_2_(OH)] and [BO_2_F_2_]) through a covertex connection, was further polymerized to form a large [B_12_O_16_F_4_(OH)_4_] cluster, which is arranged parallel to each other. Notably, the cluster with four π-conjugated [B_2_O_4_(OH)] units favors large optical anisotropy, which was verified by the response electron distribution anisotropy analysis.

**Fig. 5:**
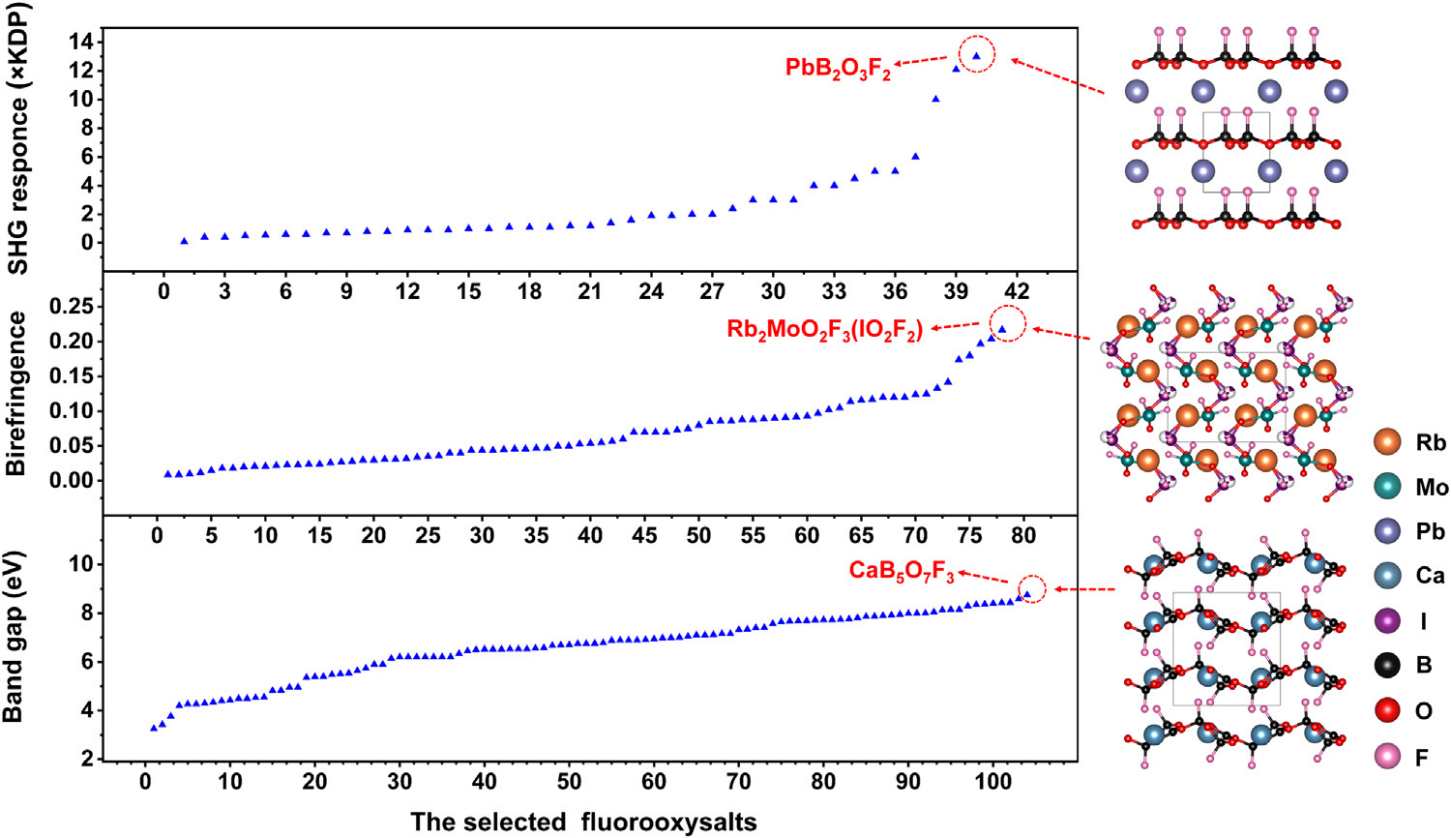
**Summary of the experimental SHG response, the birefringence and the band gap values of selected fluooxysalts stored in ICSD.** The evaluation criteria of SHG is *d*_36_ (KDP) = 0.39 pm/V. Most of the birefringence values are derived from the calculated values at 1064 nm, and the rest are derived from experimental measurements. Most of the band gap values are derived from the calculated values based on HSE06 or PBE0 methods, and the rest are derived from experimental measurements, like transmission spectrum and Tauc plot method. On the right side of the statistical diagrams are the structures of fluorooxysalts with the largest SHG effect (PbB_2_O_3_F_2_), birefringence (Rb_2_MoO_2_F_3_(IO_2_F_2_)), and band gap (CaB_5_O_7_F_3_), respectively. The selected fluooxysalts correspond to Table S2, Table S3, and Table S4, respectively.

Furthermore, alkaline earth metal fluorooxoiodate is predicted to be a possible UV birefringent crystal due to its high band gap and birefringence. By comparing the polarizability anisotropy of each [IOF] unit: *δ*(IO_4_) > *δ*(IO_3_F) > *δ*(IO_3_) > *δ*(IO_2_F_2_) > *δ*(IO_4_F) > *δ*(IO_2_F_4_), fluorooxoiodates with [IO_3_F] units are beneficial to producing large birefringence. Since the polarizability anisotropy of [IO_3_F] and [IO_3_] is very different, there is a significant gain on birefringence, such as Ba(IO_3_)F (*Δn*_cal._ = 0.125@589.3 nm) → BaI_2_O_5_F_2_ (*Δn*_cal._ = 0.174@1064 nm) and Sr(IO_3_)_2_ (*Δn*_cal._ = 0.093@1064 nm) → SrI_2_O_5_F_2_ (*Δn*_cal_*_._* = 0.203@532 nm) [43,100]. Up to now, the fluorooxoiodate Rb_2_MoO_2_F_3_(IO_2_F_2_) has possessed the largest birefringence (*Δn*_cal._ *=* 0.217@1064 nm) in fluorooxysalts as shown in Fig. 5. The anionic framework of Rb_2_MoO_2_F_3_(IO_2_F_2_) features a 0D [MoO_2_F_3_(IO_2_F_2_)] polyanion, composed of a highly distorted [MoO_3_F_3_] octahedron and a [IO_2_F_2_] group by sharing one O atom. The large birefringence mainly comes from the [IO_2_F_2_] and [MoO_3_F_3_] primitives by the real space atom-cutting method.

The physical essence of phase matching condition is to make the fundamental frequency light have the same phase when the doubling-frequency light excited at each point along the way in the crystal propagates to the outgoing plane, so that the mutual interference can be enhanced, so as to achieve high SHG efficiency. For anisotropic crystals, due to the existence of birefringence, the refractive index relationship between different polarized light can be used to achieve phase matching conditions [101]. Non-π conjugated tetrahedral units, like [BO_4_], [PO_4_], and [SO_4_] have higher HOMO-LUMO gaps than π-conjugated planar units, facilitating UV and deep-UV transmission. In addition, non-π conjugated tetrahedra avoid large in-plane anisotropy when constructing anion frameworks to favor the growth of large single crystals. It is also the case that anionic frameworks containing only rigid tetrahedral units contribute less to the birefringence, which limits the shortest phase-matching wavelength of frequency doubling crystals. To ameliorate this problem, researchers found that the introduction of fluorine could break the high symmetry of the rigid tetrahedral and the cut-off edge of the crystal was not redshifted in most cases because fluorine is more electronegative than oxygen. Fluorooxophosphate and fluorooxosulfate systems have since emerged as the go-to systems for researching the possibility of improving the associated optical properties by using fluorinated tetrahedral units. Comparing the polarizability anisotropy of the fluorinated tetrahedra to that of the original tetrahedra, theoretical calculations reveal an improvement. For example, Na_2_PO_4_ (*Δn*_cal_*_._* = 0.004 at 546 nm) → Na_2_(PO_3_F) (*Δn*_cal_*_._* = 0.023 at 546 nm); Li_2_SO_4_ (*Δn*_cal._ = 0.004 at 546 nm) → Li_2_(SO_3_F) (*Δn*_cal_*_._* = 0.057 at 546 nm); and K_2_SO_4_ (*Δn*_cal._ = 0.005 at 546 nm) → K_2_(SO_3_F) (*Δn*_cal_*_._* = 0.024 at 546 nm) [21]. In 2019, Chen's group reported a case of monofluorooxophosphate NaNH_4_PO_3_F·H_2_O, which has the shortest phase-matching wavelength among available phosphates (194 nm), achieving the ability to output deep-UV lasers [91]. In other non-centrosymmetric fluorooxophosphates, the disordered arrangement of [PO_3_F] units affects the gain of the birefringence to the extent that short phase-matching wavelengths are not possible. In NaNH_4_PO_3_F·H_2_O, the most polar P-F bonds are arranged in a nearly parallel manner, which enhances the in-plane anisotropy of (010) plane. Finding a uniform way to assemble fluorinated tetrahedra is currently crucial to investigating novel deep-UV nonlinear optical crystals in non-π-conjugated systems. Interestingly, some non-centrosymmetric fluorooxophosphates with a smaller birefringence can achieve the output of a UV laser by the quadruple frequency, which is difficult to achieve in π-conjugated systems, like (NH_4_)_2_PO_3_F (*Δn*_cal_*_._* = 0.035 at 532 nm, 0.2 × BBO) and [C(NH_2_)_3_]_2_PO_3_F (*Δn*_cal_*_._* = 0.039 at 532 nm, 0.2 × BBO) [89]. In addition to birefringence, chromatic dispersion also has an influence on the phase-matching of crystals. The smaller the chromatic dispersion, the shorter the phase-matching wavelength. Therefore, the fluorooxophosphate system with small chromatic dispersion is also an excellent system for exploring deep-UV nonlinear optical crystals.

### 4.3 Band gap

The band gap in optical materials has the ability to reflect the UV cutoff edge. The UV cutoff edge gets shorter with increasing band gap. Rekindled interest in the creation of deep-UV nonlinear optical and birefringent crystals, crucial constituents of solid-state lasers functioning in this spectral range, has resulted from the growing need for deep-UV laser sources. Deep-UV transmission requires crystals to have a band gap larger than 6.2 eV. The localized molecular orbitals of anionic units and the corresponding localized ionic orbitals of cations are often responsible for determining the absorption edge of inorganic crystals. For deep-UV transparent crystals, in the absence of cations with *d*-*d* and *f*-*f* electron transitions, the band gap is mainly affected by anionic groups, like B-O, C-O, P-O, S-O, N-O, and Si-O groups. As mentioned earlier, π-conjugated units have higher polarizability anisotropy and hyperpolarizability than non-π conjugated units, showing large SHG response and birefringence. On the other hand, the UV cutoff edge is redshifted due to a tiny HOMO-LUMO gap of π-conjugated units caused by dangling bonds. Researchers have discovered that by joining terminal oxygen to a covalent tetrahedron, dangling bonds in materials can be successfully removed. At present, no covalent tetrahedra have been found to be connected with [CO_3_] and [NO_3_] in nitrate and carbonate systems. However, covalent tetrahedra are commonly observed in borates, where they play an important role in the structural and functional properties of these materials. These covalent tetrahedra include pure oxidized tetrahedra ([BO_4_], [BeO_4_], [AlO_4_], and [ZnO_4_]) and fluorinated tetrahedra ([BeO_3_F], [AlO_3_F], and [ZnO_3_F]) [88]. Like covalent tetrahedra in borates, fluorinated [BOF] units can be linked to [BO_3_] and [B_3_O_6_] units to remove (or partly remove) dangling bonds and improve the electronic properties of borate materials. For example, fluorooxoborate CsB_4_O_6_F and borate β-BBO share [B_3_O_6_] units in their structures; however, CsB_4_O_6_F has a much lower UV cutoff edge (155 nm) than β-BBO (189 nm) due to the presence of bridging [BO_3_F] units [102]. The fluorooxoborate CaB_5_O_7_F_3_ has the largest band gap (cal. 8.75 eV) in fluorooxysalts. Structurally, the FBB of CaB_5_O_7_F_3_ [38], [B_5_O_9_F_3_], composed of three [BO_3_F] and two [BO_3_] units, connects to each other to form ^2^[B_5_O_7_F_3_]_∞_ layers along the *ac* plane. The dangling bonds of each [BO_3_] are eliminated by fluorinated [BO_3_F] units, resulting in a large band gap. The partial HOMO-LUMO gap of fluorinated groups such as [BOF] and [POF] has been found to be similar to that of pure oxygen tetrahedra. This suggests that when an anion contains only fluorinated tetrahedra, the UV cutoff edge of the crystals may shift towards the deeper UV region. In some cases, this shift can result in UV cutoff edges equivalent to or even shorter than those obtained with other nonlinear optical crystals. For example, strontium tetraborate (SrB_4_O_7_) [102], [103], [104] has a UV cutoff edge of 120 nm, which is the shortest transmission wavelengths observed using a second-order nonlinear optical process in a solid-state crystal. In sulfate and sulfite systems, the incorporation of fluorinated groups such as [SO_3_F] and [SO_2_F] can negatively impact the band gap energy of the material (see Table 1). The HOMO-LUMO gap of [SO_3_F] and [SO_2_F] has been found to be lower than that of [SO_4_] and [SO_3_], resulting in a reduced band gap energy of 0.98 and 1.24 eV, respectively. This can be a significant impact on the electronic properties of the material, affecting its optical absorption, conductivity, and other properties. In the iodate system, the HOMO-LUMO gap of the [IO_4_] unit is relatively small, which limits its application in wider spectral bands. However, the incorporation of fluorinated units into the structure can significantly increase the HOMO-LUMO gap by at least 2.2 eV.

**Table 1:** **Basic electronic properties of [BO_3_], [BO_4_], [BO_3_F], [BO_2_F_2_], [BOF_3_], [SO_4_], [SO_3_], [SO_3_F], [SO_2_F], [PO_4_], [PO_3_F], [PO_2_F_2_], [IO_3_], [IO_4_], [IO_3_F], [IOF_4_], [IO_2_F_4_], and [IO_2_F_2_] anionic units, including HOMO−LUMO bandgap (*E*_g_), polarizability anisotropy (*δ*), hyperpolarizability tensor (*β_xyz_*), and largest hyperpolarizability tensor (*β*_max_).**^a^

Property	Direction	[BO_3_]	[BO_4_]	[BO_3_F]	[BO_2_F_2_]	[BOF_3_]	[SO_4_]	[SO_3_]	[SO_3_F]	[SO_2_F]
HOMO		15.95	28.8	22.82	15.93	8.77	4.8	7.12	-2.4	-0.83
LUMO		24.42	39.56	32.64	26.1	18.31	14.84	15.25	6.66	6.06
*E*_g_ (eV)		**8.48**	**10.76**	**9.82**	**10.17**	**9.54**	**10.04**	**8.13**	**9.06**	**6.89**
*δ*		**7.01**	**1.56**	**3.23**	**4.03**	**2.95**	**0.36**	**8.75**	**4.67**	**11.29**
*β_xyz_*	*xxx*	0.00	0.00	−38.04	−0.65	−7.99	−6.44	−17.20	20.60	57.78
	*xxy*	−10.79	0.00	−1.69	−6.14	16.98	−1.60	−19.88	−0.51	0.00
	*xyy*	0.00	0.00	−25.49	−3.70	−2.23	0.23	17.23	−7.51	35.51
	*yyy*	10.8	0.00	6.53	−48.15	32.01	9.14	20.05	−6.25	0.00
	*xxz*		3.61	0.00	−3.62	0.00	−2.08	−39.72	0.00	5.79
	*xyz*	0.00	0.00	0.00	1.57	0.00	−0.04	−0.04	0.00	0.00
	*yyz*		−3.61	0.00	−16.75	0.00	−2.54	−39.82	0.00	12.62
	*xzz*	0.00	0.00	−25.74	1.63	−17.96	5.11	0.02	−7.87	9.18
	*yzz*	−0.01	0.00	−7.79	−9.69	12.51	−2.27	0.13	2.79	0.00
	*zzz*		0.00	0.00	−13.61	0.00	−0.29	−68.48	0.00	−40.85
*β_max_*		**10.80**	**3.61**	**−38.00**	**−48.15**	**32.01**	**9.14**	**−68.48**	**20.60**	**57.78**

Property	Direction	**[PO_4_]**	**[PO_3_F]**	**[PO_2_F_2_]**	**[IO_3_]**	**[IO_4_]**	**[IO_3_F]**	**[IOF_4_]**	**[IO_2_F_4_]**	**[IO_2_F_2_]**

HOMO		11.91	5.31	−1.91	−1.68	11.94	4.01	−3.87	13.48	−2.82
LUMO		20.84	14.12	7.45	3.77	14.94	9.27	3.24	18.74	3.33
*E*_g_ (eV)		**8.93**	**8.81**	**9.36**	**5.45**	**3.00**	**5.26**	**7.11**	**5.26**	**6.15**
*δ*		**0.55**	**5.04**	**5.08**	**15.33**	**34.01**	**22.26**	**11.08**	**9.78**	**14.67**
*β_xyz_*	*xxx*	−7.64	−3.25	0.33	59.92	−30.84	−93.36	0.00	26.09	−37.40
	*xxy*	−13.00	−11.32	0.00	−20.81	−11.58	43.55	−11.76	1.83	−4.45
	*xyy*	−0.06	3.18	−0.91	17.26	−47.35	−75.76	0.00	3.03	12.30
	*yyy*	−7.92	17.71	0.00	−64.89	8.73	−12.85	−9.73	−6.88	−36.22
	*xxz*	1.70	1.96	9.98	2.34	58.17	−20.15	−6.91	15.75	−10.78
	*xyz*	3.02	0.05	0.00	−4.06	−6.00	14.23	0.00	1.49	−2.85
	*yyz*	0.46	2.92	−8.21	0.66	27.10	−25.21	11.20	8.59	19.75
	*xzz*	−2.20	−2.59	−1.61	13.81	−20.94	−8.67	0.00	7.27	−17.61
	*yzz*	10.44	−10.64	0.00	−22.05	−7.34	−22.07	23.99	−14.88	−18.42
	*zzz*	3.51	−4.23	−26.07	30.97	115.07	−52.07	19.23	30.98	48.50
*β_max_*		**−13.00**	**17.71**	**−26.07**	**−64.89**	**115.07**	**−93.36**	**23.99**	**30.98**	**48.50**

a These values were calculated by building the corresponding geometric models of [BO_3_], [BO_4_], [BO_3_F], [BO_2_F_2_], [BOF_3_], [SO_4_], [SO_3_], [SO_3_F], [SO_2_F], [PO_4_], [PO_3_F], [PO_2_F_2_], [IO_3_], [IO_4_], [IO_3_F], [IOF_4_], [IO_2_F_4_] and [IO_2_F_2_] units from Kbe_2_BO_3_F_2_ (*R*32), AsBO_4_ (*I*$\bar{4}$), SrB_5_O_7_F_3_ (*Cmc*2_1_), BaBOF_3_ (*P*2_1_/c), BaBOF_3_ (*Pnma*), LiNH_4_SO_4_ (*Pca*2_1_), Na_2_SO_3_ (*P*$\bar{3}$), LiSO_3_F (*C*2/*m*), RbSO_2_F (*P*2_1_/*m*), Na_3_PO_4_ (*P*$\bar{4}$2_1_*c*), Na_2_PO_3_F (*P*2_1_2_1_2_1_), NH_4_PO_2_F_2_ (*Pnma*), Sr(IO_3_)_2_ (*P*$\bar{1}$), Bi_2_(IO_4_)(IO_3_)_3_ (*P*2_1_2_1_2_1_), SrI_2_O_5_F_2_ (*P*2_1_/*c*), CsIOF_4_ (*Pnma*), SbF_5_IF_3_O_2_ (*P*2_1_/*c*), and Ba(IO_2_F_2_)_2_ (*P*2_1_/*c*), respectively.

## 5 Other properties and applications of fluorooxysalts

### 5.1 Electrolyte additive for ionic batteries

**Fig. 6:**
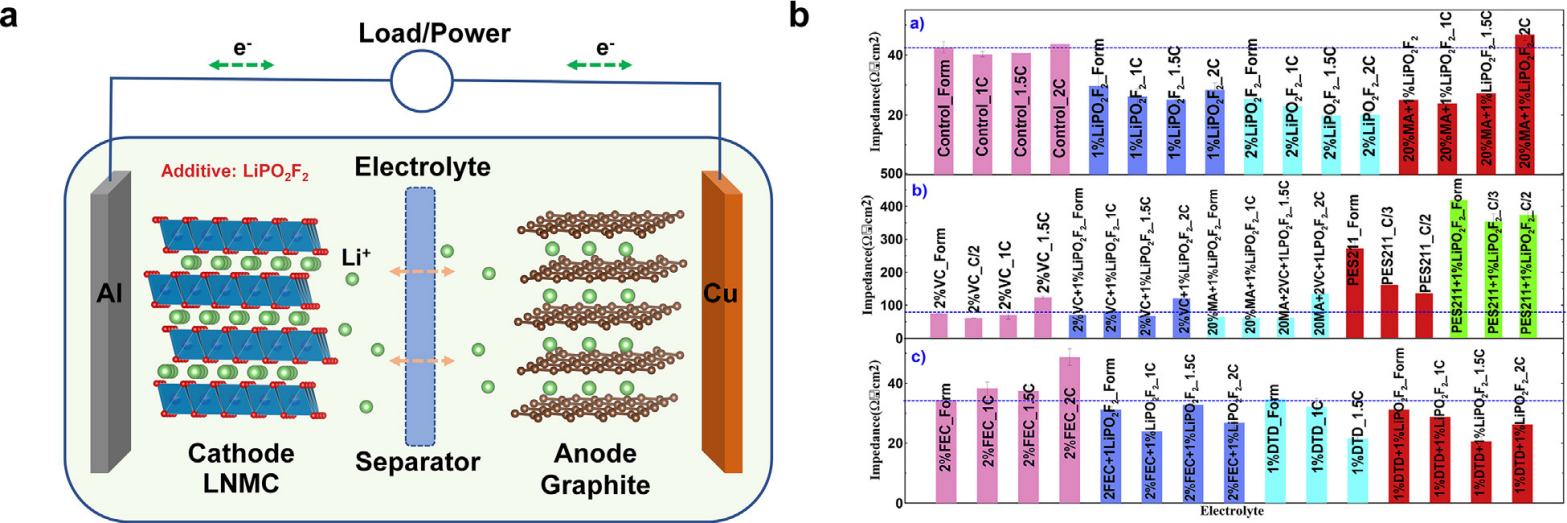
(a) Working principles of lithium ion batteries. (b) The diameter of the semicircle in the EIS spectra for NMC111/graphite pouch cells, after cell formation and after 20°C cycling at C/3, C/2, 1C, 1.5C and 2C for cells containing a) control, control + 1% LiPO_2_F_2_, control + 2% LiPO_2_F_2_, 1.2 M LiPF_6_ in EC:EMC (3:7) and 1.2 M LiPF_6_ in [20% MA+ 80% EC:EMC (3:7)] + 1% LiPO_2_F_2_; b) 2% VC, 2% VC +1% LiPO_2_F_2_, PES211 and PES211 + 1% LiPO_2_F_2_ and c) 2% FEC, 2% FEC + 1% LiPO_2_F_2_, 1% DTD and 1% DTD + 1% LiPO_2_F_2_. Reproduced with permission from ref. [108] Copyright 2018 Elsevier.

The demand for mobile power solutions has increased as a result of the extensive usage of portable electronics and mobile gadgets in the electronic information era [105], [106], [107], [108], [109]. Owing to the significant benefits of high voltage, high capacity, long cycle life, and good safety performance, lithium-ion batteries have become a hot topic for research in recent years and have a wide range of applications in the national defense industry, electric vehicles, portable electronic equipment, and space technology [105], [106], [107], [108], [109]. The graphite potential in a lithium-ion battery cell may shift below the lithium potential if there is adequate polarization, which could result in unintended lithium plating on the graphite electrode [105]. When lithium ion cells are subjected to high charge rates and/or low temperatures, undesired lithium plating becomes the predominant aging process due to the detrimental effect of this deposited lithium on the cells' lifetime. Making the right electrolyte additive selections is crucial to solving this issue [106]. For the past few years, a fluorooxysalt, lithium difluorooxophosphate LiPO_2_F_2_ has been found to be a promising electrolyte additive [107]. With the electrochemical impedance spectroscopy (EIS), Dahn et al. found that cells with 1% LiPO_2_F_2_ have outstanding storage performance at 60°C [108]. The behavior of LiPO_2_F_2_ depends on the presence of other additives. When 1% LiPO_2_F_2_ was combined with 1% DTD or 2% FEC, impedance growth during cycling was highly suppressed although the cycle performance was not improved for the cells used here (Fig. 6b). Kim et al. demonstrated that combining LiPO_2_F_2_ with the organic additive vinylene carbonate (VC) can effectively modify the properties of the electrode/electrolyte interface to increase electron/ion transport rate, thereby significantly improving the charge/discharge performance of NMC/graphite cells. Yang et al. present LiPO_2_F_2_ as a salt-type electrolyte additive to enhance the cycling stability of large-size crystallite LiNi_0.5_Mn_0.25_Co_0.25_O_2_ (LNMC) cathodes (Fig. 6a) [109]. Results demonstrate that 1 wt% LiPO_2_F_2_ can significantly improve not only the initial coulombic efficiency by 3%, but also the cycling stability and rate capability at 25°C. In order to study the working mechanism of LiPO_2_F_2_ on LNMC electrode, relevant theoretical calculations were also carried out. In the optimized structure of LiPO_2_F_2_, the Li atom coordinates with one of the oxygen atoms in equidistance, resulting in a stable state in the solvents. Another oxygen atom in the LiPO_2_F_2_ structure may be coordinates with Li^+^ in electrolyte; this is the reason why adding LiPO_2_F_2_ in the electrolyte can decrease the conductivity. It may also anchor on the cathode surface combining with Li^+^ on the first surface layer of the cathode (or positive) electrode at the same time. The theoretical results also indicate that the dissociation energy between Li^+^ and [PO_2_F_2_] is stronger than those energy of Li^+^ bonding with [ClO_4_], indicating that LiPO_2_F_2_ is hard to dissolve and dissociate in the non-aqueous media, which causes the enrichment of LiPO_2_F_2_ on the surface of cathode to form the protective cathode surface at the beginning of cycling process.

### 5.2 Inorganic framework materials

A family of materials known as framework materials is made up of linked polyhedra or other building blocks. Because of their potential uses in a variety of industries, such as sorption, ion exchange, energy storage, and catalysis, framework materials have attracted a lot of attention recently [110]. Because of the framework structure's interconnectivity, guest species can be incorporated into the material's pores and channels, facilitating the uptake and release of molecules or ions and opening up possibilities for uses like gas storage and separation. Fluorine addition to metal phosphate framework structures can modify the chemistry of the material in both structural and compositional ways [110]. The local coordination environment and bonding interactions within the framework structure can alter when fluorine is substituted for oxygen on a metal or phosphorus site. These changes can then impact the electrical, optical, and catalytic properties of the material. For example, Lü et al. studied the antiferromagnetism of Ba_2_M_2_(PO_3_F)F_6_ (M = Mn, Co, Ni and Cu) series [111], and found that the arrangement of 0D metal chains can be regulated by changing the cations in the case of the same *cis*-MO_2_F_4_ metal polyhedra and [PO_3_F] tetrahedra, thus displaying different antiferromagnetism. Weller et al. synthesized a series of transition metals fluorooxophosphates by hydrofluorination and preliminarily characterized their properties, like reductive lithium ion insertion, ion exchange reactions and magnetic susceptibility behaviors [42]. Despite the significant advancements that fluorooxysalts have made in the field of materials science, there are still some unanswered questions. The most important one of these is figuring out where OH^−^ and F^−^ reside in the structures. For your reference, we shall then go over the procedures and developments of these means.

## 6 Identification of OH/F in fluorooxysalts

**Fig. 7:**
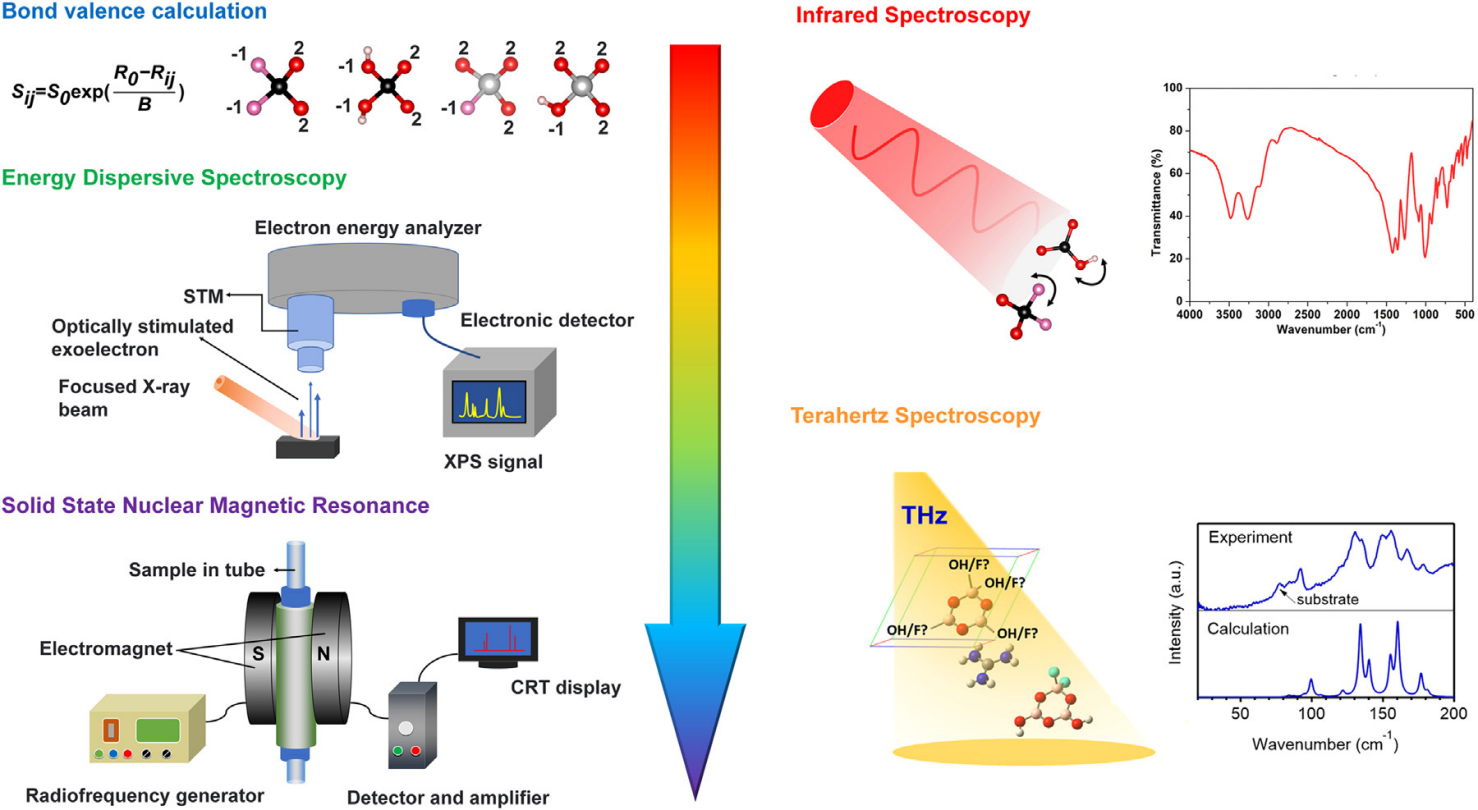
**Current methods for the identification of hydroxyl and fluorine in fluorooxysalts.** Reproduced with permission from refs. [99] and [121]. Copyright 2022 Wiley and Copyright 2023 The American Chemical Society.

It is difficult to identify the existence of OH^−^ and F^−^ in the process of synthesis of fluorooxysalts only based on the electron density pattern of XRD single crystal diffraction. Because the chemical coordination environment of OH^−^ and F^−^ is similar and the elements are relatively light. Therefore, a variety of theoretical and experimental characterization methods have been used to identify OH^−^ and F^−^ anions. **(1) Empirical judgment based on inorganic chemistry.** Using information from previous studies and experimental observations, empirical judgment based on inorganic chemistry entails forecasting or drawing judgments about the behavior of inorganic substances. In the fluorooxoborate system, the existence of triangular [BO_2_F] units has not been confirmed experimentally, and tri-coordinated boron is typically observed as [BO_2_(OH)] in the presence of both OH^−^ and F^−^ ions. This is because the electronegativity of fluorine is greater than that of oxygen, which makes it more difficult for F^−^ to bond with B^3+^ cations and then it will form stable [BO_2_F] units compared to [BO_2_(OH)] units. **(2) Bond valence calculation (BVS).** Pauling's rule of electroneutrality states that in a stable ionic compound, the sum of the positive charges (i.e. cationic charges) must be equal to the sum of the negative charges (i.e. anionic charges) [112]. According to this rule, the electronegativity difference between cations and anions determines the strength of ionic bonds. Therefore, the bond strengths from neighboring cations to the anion are approximately equal to the electronegativity difference between the two ions. Based on this, Brown improved and gave the relationship between bond valence and bond length: $S_{ij}=S_{0}\text{exp}(\frac{R_{0}-R_{ij}}{B})$ [113]. The crystallographic position of OH/F can be preliminarily determined by BVS. **(3) Energy dispersive spectroscopy (EDS).** The spectrometer analyzes the type and content of elements in the microcomponent of materials based on the characteristic energy of X-ray photons of various elements [114]. The presence or non-presence of F can be determined on the spectrogram when the micron-sized crystals are scanned multiple times. **(4) Infrared spectrum.** In order to determine the type of chemical bonds or functional groups present in a molecule, different chemical bonds or functional groups will absorb at different frequencies and appear in distinct locations in the infrared spectrum [115]. The vibrations of OH^−^ and most B/P/S/I-F bonds can be seen in infrared spectroscopy. **(5) The nuclear magnetic resonance spectroscopy.** When the irradiation frequency is determined, the phenomenon that the same nucleus shows absorption peak under different resonance magnetic field intensity because of different chemical environment in the molecule is called chemical shift [116]. The atoms in different chemical coordination environments can be distinguished by the relative chemical shifts and coupling constants of different spectra. As early as 1969, Farrar et al. studied the chemical shifts and coupling parameters of ^31^P and ^19^F for BaPO_3_F [117]. At present, magic angle rotation solid-state nuclear magnetic is used to identify OH^−^ and F^−^ for fluorooxysalts. **(6) Terahertz (THz) spectroscopy.** THz spectroscopy has emerged as a new tool for crystal structure analysis. In organic molecules, ligand substitution leads to differences in molecular interactions, which lead to differences in physical properties and leave different fingerprint peaks in the terahertz spectrum [118]. Thus, THz spectroscopy studies intermolecular interactions on a much broader scale than NMR and IR spectroscopy, which analyze intermolecular magnetic fields and interactions. Zhang et al. have elucidated the power of THz spectroscopy to address several critical issues in X-ray crystallography. For example, they used THz spectroscopy to resolve hydrogen atoms that are invisible to X-ray [119], and they determined the microscopic structures induced by atomic occupational disorder and molecular orientational disorder, respectively, whose information is difficult to be retrieved by diffraction methods [120]. Recently, THz spectroscopy has been proved to be an effective means to identify OH^−^ and F^−^ in structures [121]. By comparing the terahertz fingerprint peaks of 16 different configurations of [B_3_O_3_(F, OH)_4_] anions in GBF compound calculated by density functional theory, the terahertz spectra characterized by experiments are in good agreement with one of them, and in agreement with the resolved single crystal structure (Fig. 7).

## 7 Conclusion and outlook

Fluorooxysalts have garnered increasing attention in the past few decades due to their rich structural chemistry and exceptional characteristics, which stem from the application of mixed anionic approach in solid-state chemistry and materials research. Birefringent crystals, framework materials, electrolyte additives, and nonlinear optics are just a few of the uses for these materials. In order to completely appreciate the potential of fluorooxysalts for the development of advanced materials with customized properties and uses, more research must be done on the structure-performance relationship, synthesis techniques, structural chemistry, and key properties, particularly optical ones. Here, we looked at the latest developments, current situation, upcoming prospects, and difficulties pertaining to the material and chemical properties of fluorooxysalts including fluorine-containing M-F bonds. Fluorooxysalts have a wider development promise than oxysalts, which is a larger and more developed field; yet, there are obstacles to overcome.

In order to keep fluorine from escaping during the synthesis of many fluorooxysalts, an enclosed environment is usually needed. This might make it challenging to monitor the reaction's progress and evaluate the quality of the crystals that are produced. The synthesis of alkali metal fluorooxoborates and fluorooxoborophosphates is the primary focus of research on fluorooxysalts in solid-state synthesis at the moment. The conventional borates and borooxooxophosphates, which have certain crystal structures and well-established synthesis procedures, are comparable to these two systems. As a result, comparable synthetic methods can be used to their fluorinated equivalents more easily, providing for greater control over the synthesis procedure and the final crystal quality. In comparison to alkaline earth metal salts, alkali metal fluorooxoborates and fluorooxoborophosphates have lower melting points. Because the reaction can take place at lower temperatures thanks to this characteristic, they are more suited for synthesis. Because of their high melting temperatures, fluorooxysalts can be difficult to synthesize using solid-state synthesis when combined with alkaline earth metals or other high melting point metals. Future study may focus on identifying an appropriate catalyst or flux to lower the reaction's activation energy. The rivalry between OH- and F-ions in the production of fluorooxysalts can be influenced by the pH of the solution and the quantity of fluorine present in hydrothermal and solvothermal reactions.

In contrast to solid-state reactions, hydrothermal processes necessitate taking into account other variables such the solution's pH, the raw materials' solubility, and the impact of added OH^−^ and H_2_O on the thermal stability. The stability of intermediate species and the reaction pathway in hydrothermal reactions can be affected by the pH of the solution. Furthermore, the behavior of crystal formation and the velocity of reaction can be influenced by the solubility of the raw materials. The addition of OH^−^ and H_2_O to the final product may decrease its thermal stability, which could have a negative impact on its intended uses. This is due to the fact that OH^−^ and H_2_O can induce faults or impurities to occur within the crystal lattice, which can affect its characteristics and performance. In spite of these difficulties, hydrothermal reactions are superior than solid-state reactions in a number of ways, such as the capacity to regulate crystal morphology and size, strengthen chemical homogeneity, and improve crystallinity. Furthermore, materials that are difficult or impossible to create through traditional synthetic pathways can be synthesized using hydrothermal processes. In general, effective synthesis procedures for fluorooxysalts and other advanced materials require a grasp of the benefits and constraints of hydrothermal processes.

It is worth investigating the mutual repulsion between fluorinated groups in other fluorooxysalts as there aren't many anionic frameworks with ≥ 1D configuration in other fluorooxysalts, other from fluorooxoborate and its mixed salts. Thus far, the only way to join fluorinated tetrahedra and polyhedra in anionic frameworks is through vertex connections, with the exception of [AsOF] and [TeOF] units. It is unknown if other fluorooxysalts have an edge-sharing or face-sharing anion group, similar to the existence of edge-shared [BO_4_] tetrahedral in borate [1]. In some oxysalts, anions that contain OH^−^ are similar to fluorinated anions, such as [BO_3_(OH)] and [BO_3_F], [HPO_4_] and [PO_3_F], [H_2_PO_4_] and [PO_2_F_2_], as well as [HSO_4_] and [SO_3_F]. However, in π-conjugated systems, no fluorinated unit has been found so far, although there are anions with OH^−^, like hydroxylated [BO_2_(OH)] and [CO_2_(OH)] units. The existence of anions such as [BO_2_F], [BOF_2_], and [CO_2_F] still needs further exploration and attempts.

Certain fluorooxysalts have demonstrated exceptional optical performance in the UV and deep-UV range. Verification is still required for the microscopic optical characteristics of fluorinated groups that the first-principles calculations yielded. Similar to the fluorooxoborate family, all three fluorinated groups show hyperpolarizability values greater than the π-conjugated [BO_3_] unit. This suggests that fluorinated units are highly promising for augmenting the second-order nonlinear optical response of various materials. Unfortunately, there are very few non-centrosymmetric compounds in the fluorooxoborate system that exclusively include fluorinated units. As a result, it is difficult to completely grasp the structure-property correlations of these compounds and research their SHG response. The arrangement of fluorinated motifs in non-π conjugated systems can significantly affect their optical characteristics, such as their birefringence and SHG response. The orientation and arrangement of fluorinated units within the crystal lattice determines their polarizability anisotropy and hyperpolarizability. The ability of the fluorinated units to contribute to the materials' total nonlinear optical response may be limited if they are organized adversely.

Strategies have been shown to be effective to partially regulate the arrangement of π-conjugated [BO_3_] units in borates by introducing the covalent tetrahedra [Be/Al/ZnO_4_] or [B/Be/Al/ZnO_3_F]. However, research on strategies for controlling non-π conjugated motif organization is scarce. Additionally, because it controls how atoms and molecules are arranged within the crystal lattice, a material's internal structure is essential to understanding both its performance and its attributes. Ensuring that the material displays the desired qualities and performs as planned for its intended application depends on the accuracy of its internal structure. All of the existing techniques for identifying OH^−^ and F^−^ anions rely on X-ray powder or single crystal diffraction data, which takes a lot of time and resources, particularly when high accuracy is needed. Future technological advancements might make it feasible to quickly determine the OH^−^ and F^−^ groups in materials at the atomic level. Furthermore, by examining the enormous quantities of spectroscopic data and finding patterns and correlations within the data, developments in computer techniques and machine learning algorithms may make it easier to quickly discover OH^−^ and F^−^ groups in materials.

## Declaration of competing interest

The authors declare that they have no conflicts of interest in this work.

## Appendix Supplementary materials

Image, application 1
