# Supplementary material for: Fluorination strategy toward chemical and functional modification
Source: Fundam Res. 2023 Dec 20;5(2):640–53. doi: 10.1016/j.fmre.2023.08.017 (PMC11997599; doi:10.1016/j.fmre.2023.08.017)
Supplement: Supplementary file 1 [file mmc1.docx]

**Fluorination strategy toward chemical and functional modification**

Haotian Qiu^a,b^, Shilie Pan^a,b,^* and Miriding Mutailipu^a,b,^*

*^a^Research Center for Crystal Materials, Xinjiang Technical Institute of Physics and Chemistry, Chinese Academy of Sciences, Urumqi 830011, People’s Republic of China*

*^b^Center of Materials Science and Optoelectronics Engineering, University of Chinese Academy of Sciences, Beijing 100049, People’s Republic of China*

** Corresponding authors: miriding@ms.xjb.ac.cn (Miriding Mutailipu) and slpan@ms.xjb.ac.cn (Shilie Pan).*

**Table S1.** Summary of fluoroxysaltes included in ICSD, CCDC and COD.

| **No.** | **Formula** | **Space group** | **Space group number** | **Symmetry** | **The anionic dimension** | **ICSD code** |
| --- | --- | --- | --- | --- | --- | --- |
| **Fluorooxoborates** | | | | | | |
| **1** | NH_4_B_4_O_6_F | *Pna*2_1_ | 33 | NCS | 2D | 254160 |
| **2** | NH_4_B_6_O_9_F_2_ | *P*2_1_*/c* | 14 | CS | 2D | 7130840^*^ |
| **3** | LiB_6_O_9_F | *Pna*2_1_ | 33 | NCS | 2D | 420286 |
| **4** | Li_2_B_6_O_9_F_2_ | *Cc* | 9 | NCS | 3D | 423435 |
| **5** | Li_2_B_3_O_4_F_3_ | *P*2_1_2_1_2_1_ | 18 | NCS | 1D | 423661 |
| **6** | Na_3_B_3_O_3_F_6_ | *C*2*/c* | 15 | CS | 0D | 424505 |
| **7** | Na_2_B_6_O_9_F_2_ | *P*2_1_*/c* | 14 | CS | 2D | 268287 |
| **8** | NaB_4_O_6_F | *C*2 | 5 | NCS | 2D | 112376 |
| **9** | Na_3_B_7_O_11_F_2_ | *Pnma* | 53 | CS | 2D | 234745 |
| **10** | Na_4_B_8_O_9_F_10_ | *Pna*2_1_ | 33 | NCS | 1D | / |
| **11** | K_3_B_3_O_3_F_6_ | *P*2_1_*/n* | 14 | CS | 0D | 251593 |
| **12** | K_3_B_6_O_9_F_3_ | *P*2_1_*/c* | 14 | CS | 0D | 130172 |
| **13** | K_10_B_13_O_15_F_19_ | *R*3*m* | 160 | NCS | 0D | 131753 |
| **14** | K_2_B_6_O_9_F_2_ | *Pbca* | 61 | CS | 2D | 7130839^*^ |
| **15** | K_2_B_3_O_4_F_3_ | *Pbcn* | 60 | CS | 1D | 45037 |
| **16** | RbB_4_O_6_F | *Pna*2_1_ | 33 | NCS | 2D | 433804 |
| **17** | Rb_3_B_3_O_3_F_6_ | *P*2_1_*/n* | 14 | CS | 0D | 11500 |
| **18** | RbB_3_O_4_F_2_ | *P*2_1_*/c* | 14 | CS | 1D | 7127602^*^ |
| **19** | Rb_10_B_13_O_15_F_19_ | *R*3*m* | 160 | NCS | 0D | 131752 |
| **20** | CsB_4_O_6_F | *Pna*2_1_ | 33 | NCS | 2D | / |
| **21** | Cs_3_B_3_O_3_F_6_ | *Pbcn* | 60 | CS | 0D | 133758 |
| **22** | Cs_4_B_4_O_3_F_10_ | *P*2_1_*/c* | 14 | CS | 0D | 135114 |
| **23** | CsRbB_8_O_12_F_2_ | *P*$\overline{\text{6}}$2*c* | 190 | NCS | 2D | 433806 |
| **24** | CsKB_8_O_12_F_2_ | *P*321 | 150 | NCS | 2D | 433805 |
| **25** | Li_2_(Na_0_._9_K_0_._1_)B_5_O_8_F_2_ | *Pbcn* | 60 | CS | 2D | 244622 |
| **26** | K_1_._66_Rb_1_._34_B_3_O_3_F_6_ | *P*2_1_*/c* | 14 | CS | 0D | 11506 |
| **27** | K_2_._64_Cs_0_._36_B_3_O_3_F_6_ | *P*2_1_*/c* | 14 | CS | 0D | 11505 |
| **28** | Cs_1_._29_Rb_1_._71_B_3_O_3_F_6_ | *P*2_1_*/c* | 14 | CS | 0D | 11504 |
| **29** | K_2_RbB_3_O_3_F_6_ | *Pbcn* | 60 | CS | 0D | 11503 |
| **30** | KCs_2_B_3_O_3_F_6_ | *P*2_1_*/c* | 14 | CS | 0D | 11502 |
| **31** | Na_0_._76_Rb_2_._24_B_3_O_3_F_6_ | *P*2_1_*/n* | 14 | CS | 0D | 11501 |
| **32** | NaKB_6_O_9_F_2_ | *P*2_1_*/c* | 14 | CS | 2D | 143683 |
| **33** | NaRbB_3_O_4_F_3_ | *P*2_1_*/c* | 14 | CS | 1D | 2086537^#^ |
| **34** | KNaB_3_O_4_F_3_ | *Pbcn* | 60 | CS | 1D | 45038 |
| **35** | KCsB_3_O_4_F_3_ | *Pbcn* | 60 | CS | 1D | 45039 |
| **36** | CsKB_8_O_12_F_2_·CsI | *R*32 | 155 | NCS | 2D | 2152178^#^ |
| **37** | CsNH_4_B_8_O_12_F_2_·CsI | *R*32 | 155 | NCS | 2D | 2152179^#^ |
| **38** | MgB_5_O_7_F_3_ | *Cmc*2_1_ | 36 | NCS | 2D | / |
| **39** | CaB_5_O_7_F_3_ | *Cmc*2_1_ | 36 | NCS | 2D | 264388 |
| **40** | CaB_4_O_6_F_2_ | *P*$\overline{\text{1}}$ | 2 | CS | 2D | 434028 |
| **41** | SrB_4_O_6_F_2_ | *P*$\overline{\text{1}}$ | 2 | CS | 2D | 434029 |
| **42** | SrB_5_O_7_F_3_ | *Cmc*2_1_ | 36 | NCS | 2D | 263469 |
| **43** | BaB_4_O_6_F_2_ | *P*2_1_*/n* | 14 | CS | 2D | 433589 |
| **44** | BaB_8_O_12_F_2_ | *R*$\overline{\text{3}}$*c* | 167 | CS | 2D | 1868310^#^ |
| **45** | BaB_2_O_3_F_2_ | *P*2_1_ | 4 | NCS | 2D | 1845846^#^ |
| **46** | BaB_5_O_8_F·xH_2_O | *Pbca* | 61 | CS | 3D | 7049873^*^ |
| **47** | Ba_2_B_7_O_12_F | *C*2*/c* | 15 | CS | 3D | 143589 |
| **48** | α-BaB_4_O_5_F_4_ | *P*2_1_ | 4 | NCS | 1D | 143444 |
| **49** | β-BaB_4_O_5_F_4_ | *P*2_1_*/c* | 14 | CS | 1D | 143445 |
| **50** | α-BaBOF_3_ | *P*2_1_ | 4 | NCS | 1D | 114121 |
| **51** | β-BaBOF_3_ | *P*2_1_*/c* | 14 | CS | 1D | 263824 |
| **52** | γ-BaBOF_3_ | *P*2_1_*/c* | 14 | CS | 1D | 114122 |
| **53** | Ba_3_B_10_O_17_F_2_·0.1KF | *P*$\overline{\text{1}}$ | 2 | CS | 2D | 7123776^*^ |
| **54** | Ca_0_._9_Mg_0_._1_B_5_O_7_F_3_ | *Cmc*2_1_ | 36 | NCS | 2D | 135972 |
| **55** | Ca_0_._88_Mg_0_._12_B_5_O_7_F_3_ | *Cmc*2_1_ | 36 | NCS | 2D | 135971 |
| **56** | Ca_0_._925_Mg_0_._075_B_5_O_7_F_3_ | *Cmc*2_1_ | 36 | NCS | 2D | 135970 |
| **57** | Ca_0_._93_Mg_0_._07_B_5_O_7_F_3_ | *Cmc*2_1_ | 36 | NCS | 2D | 135969 |
| **58** | Ca_0_._92_Mg_0_._08_B_5_O_7_F_3_ | *Cmc*2_1_ | 36 | NCS | 2D | 135968 |
| **59** | Ca_0_._94_Mg_0_._06_B_5_O_7_F_3_ | *Cmc*2_1_ | 36 | NCS | 2D | 135967 |
| **60** | BiB_2_O_4_F | *P*3_2_ | 145 | NCS | 1D | 172481 |
| **61** | PbB_2_O_3_F_2_ | *P*31*m* | 157 | NCS | 2D | 263596 |
| **62** | PbB_5_O_8_F | *Pbca* | 61 | CS | 3D | 256206 |
| **63** | PbB_5_O_7_F_3_ | *Cmc*2_1_ | 36 | NCS | 2D | 142090 |
| **64** | SnB_2_O_3_F_2_ | *P*31*m* | 157 | NCS | 2D | 263597 |
| **65** | KNiB_4_O_6_F_3_ | *P*2_1_*/c* | 14 | CS | 2D | 1926089^#^ |
| **66** | Na[(UO_2_)B_5_O_8_(OH)F]3H_2_O | *Cc* | 9 | NCS | 2D | 182938 |
| **67** | K[(UO_2_)B_5_O_8_(OH)F] | *P*2_1_*/n* | 14 | CS | 2D | 182939 |
| **68** | K_11_[(UO_2_)_6_B_24_O_36_F_22_)](H_2_BO_3_) | *P*6_3_*/m* | 176 | CS | 2D | 182940 |
| **69** | Rb[(UO_2_)B_5_O_8_(OH)F] | *Cc* | 9 | NCS | 2D | 182941 |
| **70** | Tl[(UO_2_)B_5_O_8_(OH)F] | *P*1 | 1 | NCS | 2D | 182942 |
| **71** | Na[(NpO_2_)B_5_O_8_(OH)F]3H_2_O | *Cc* | 9 | NCS | 2D | 182943 |
| **72** | Rb_2_[(UO_2_)_2_B_8_O_12_F_6_]·H_2_B_4_O_7_ | *P*1 | 1 | NCS | 2D | 868274^#^ |
| **73** | Na[(UO_2_)B_5_O_8_(OH)F] | *P*2_1_*/n* | 14 | CS | 2D | 868273^#^ |
| **74** | Ag[(UO_2_)B_5_O_8_(OH)F] | *P*2_1_*/n* | 14 | CS | 2D | 868271^#^ |
| **75** | Cs[(UO_2_)B_5_O_8_(OH)F] | *P*2_1_*/n* | 14 | CS | 2D | 868272^#^ |
| **76** | Na(B_3_O_3_F_2_(OH)_2_)(B(OH)_3_) | *P*$\overline{\text{1}}$ | 2 | CS | 0D | 113099 |
| **77** | Ba(B_2_OF_3_(OH)_2_)_2_ | *C*2*/m* | 15 | CS | 1D | 7125980^*^ |
| **78** | NaB_3_O_4_F(OH) | *P*2_1_*/c* | 14 | CS | 1D | 2171449^#^ |
| **79** | LiB_5_O_5_F_2_(OH)_4_ | *P*2_1_*/m* | 11 | CS | 1D | / |
| **80** | (NH_4_)_4_[B_12_O_16_F_4_(OH)_4_] | *P*4*/ncc* | 126 | CS | 0D | 2086422^#^ |
| **Fluorophosphates** | | | | | | |
| **81** | K_2_(PO_3_F) | *Pnma* | 62 | CS | 0D | 15691 |
| **82** | K(PO_2_F_2_) | *Pnma* | 62 | CS | 0D | 26640 |
| **83** | Cs(PO_2_F_2_) | *Pnma* | 62 | CS | 0D | 16875 |
| **84** | (NH_4_)(PO_2_F_2_) | *Pnma* | 62 | CS | 0D | 16152 |
| **85** | Ca(PO_3_F)·_2_H_2_O | *P*$\overline{\text{1}}$ | 2 | CS | 0D | 2802 |
| **86** | (NH_4_)_2_(PO_3_F)(H_2_O) | *P*2_1_*/c* | 14 | CS | 0D | 2803 |
| **87** | Sn(PO_3_F) | *P*2_1_*/c* | 14 | CS | 0D | 2039 |
| **88** | LiK(PO_3_F)(H_2_O) | *P*2_1_*/c* | 14 | CS | 0D | 2044 |
| **89** | Na_2_(PO_3_F) | *P*2_1_2_1_2_1_ | 19 | NCS | 0D | 2100 |
| **90** | NaK_3_(PO_3_F)_2_ | *P*$\overline{\text{3}}$*m*1 | 164 | CS | 0D | 1904 |
| **91** | Rb(PO_2_F_2_) | *Pnma* | 62 | CS | 0D | 1980 |
| **92** | Li(NH_4_)(PO_3_F) | *P*2_1_*/c* | 14 | CS | 0D | 1439 |
| **93** | K_2_(P_2_O_5_F_2_) | *C*2*/c* | 15 | CS | 0D | 1659 |
| **94** | Zn(PO_3_F)·2.5H_2_O | *P*$\overline{\text{1}}$ | 2 | CS | 0D | 35644 |
| **95** | Cu(PO_2_F_2_)_2_ | *Fddd* | 70 | CS | 0D | 61103 |
| **96** | Co(PO_3_F)·3H_2_O | *P*$\overline{\text{1}}$ | 2 | CS | 0D | 62512 |
| **97** | CuPO_3_F·H_2_O | *P*2_1_*/c* | 14 | CS | 0D | 33923 |
| **98** | Cu_2_K(OH)(PO_3_F)_2_(H_2_O) | *P*2_1_*/c* | 14 | CS | 0D | 62646 |
| **99** | (NH_4_)_2_(Ni(H_2_O)_6_)(PO_3_F)_2_ | *P*2_1_*/c* | 14 | CS | 0D | 71561 |
| **100** | (NH_4_)_2_(Cu(H_2_O)_2_(PO_3_F)_2_) | *C*2*/m* | 15 | CS | 0D | 74551 |
| **101** | K_3_(PO_3_F)F | *I*4*/mcm* | 140 | CS | 0D | 82751 |
| **102** | Cs(HPO_3_F) | *C*2*/m* | 15 | CS | 0D | 410932 |
| **103** | Cs_3_(NH_4_)_2_(HPO_3_F)_3_(PO_3_F) | *P*2_1_*/c* | 14 | CS | 0D | 410933 |
| **104** | Na(HPO_3_F)(H_2_O)_2_._5_ | *C*2*/c* | 15 | CS | 0D | 51337 |
| **105** | Na_2_(PO_3_F)(H_2_O)_10_ | *P*2_1_*/c* | 14 | CS | 0D | 51338 |
| **106** | SbF_4_(PO_2_F_2_) | *P*$\overline{\text{1}}$ | 2 | CS | 0D | 51535 |
| **107** | Rb(HPO_3_F) | *P*2_1_*/n* | 14 | CS | 0D | 151293 |
| **108** | (NH_4_)_2_(PO_3_F) | *Pna*2_1_ | 33 | NCS | 0D | 280924 |
| **109** | α-(NH_4_)(HPO_3_F) | *P*2_1_*/n* | 14 | CS | 0D | 411903 |
| **110** | β-(NH_4_)(HPO_3_F) | *P*$\overline{\text{1}}$ | 2 | CS | 0D | 411902 |
| **111** | K(HPO_3_F) | *P*2_1_ | 4 | NCS | 0D | 391178 |
| **112** | K_3_(H(PO_3_F)_2_) | *C*2*/c* | 15 | CS | 0D | 391179 |
| **113** | Hg_2_(PO_3_F) | *Ibam* | 72 | CS | 0D | 414292 |
| **114** | Rb_2_PO_3_F | *Pnma* | 62 | CS | 0D | 172343 |
| **115** | Cs_2_PO_3_F | *Pnma* | 62 | CS | 0D | 172345 |
| **116** | (NH_4_)Na(PO_3_F)(H_2_O) | *Pc* | 7 | NCS | 0D | 240974 |
| **117** | Ag_2_(PO_3_F) | *C*2*/c* | 15 | CS | 0D | 416858 |
| **118** | (NH_4_)Ag_3_(PO_3_F)_2_ | *C*2 | 5 | NCS | 0D | 172980 |
| **119** | (NH_4_)_2_(Co_3_F_2_(P_2_O_6_F)_2_) | *P*2_1_*/c* | 14 | CS | 0D | 261591 |
| **120** | (NH_4_) (MnF_2_PO_3_F) | *P*2_1_*/n* | 14 | CS | 0D | 261594 |
| **121** | (NH_4_)_2_Cu_3_F_4_P_4_O_12_ | *P*2_1_*/c* | 14 | CS | 0D | 261596 |
| **122** | Rb_2_Cu_3_F_4_P_4_O_12_ | *P*2_1_*/c* | 14 | CS | 0D | 261597 |
| **123** | KCu_3_(PO_2_F_2_)(PO_3_F)_2_F_2_ | *C*2*/c* | 15 | CS | 0D | 261598 |
| **124** | RbCu_3_(PO_2_F_2_)(PO_3_F)_2_F_2_ | *C*2*/c* | 15 | CS | 0D | 261599 |
| **125** | Cs_2_Cu_3_(PO_3_F)_4_ | *P*1 | 1 | NCS | 0D | 884286^#^ |
| **126** | CsCu_2_P_2_O_6_F_3_ | *Pnma* | 62 | CS | 0D | 884287^#^ |
| **127** | CuPO_3_F | *P*$\overline{\text{1}}$ | 2 | CS | 0D | 884288^#^ |
| **128** | K_2_Cu_3_(PO_3_F)_4_ | *P*2_1_*/n* | 14 | CS | 0D | 884289^#^ |
| **129** | KCu_3_P_3_O_10_F_4_ | *C*2*/c* | 15 | CS | 0D | 261598 |
| **130** | Na_2_Cu_2_P_2_O_7_F_2_ | *C*2*/c* | 15 | CS | 0D | 884291^#^ |
| **131** | Rb_2_Cu_3_(PO_3_F)_4_ | *P*2_1_*/c* | 14 | CS | 0D | 261597 |
| **132** | RbCu_3_P_3_O_6_F_8_ | *C*2*/c* | 15 | CS | 0D | 261599 |
| **133** | (NH_4_)_2_Cu_3_(PO_3_F)_4_ | *P*2_1_*/c* | 14 | CS | 0D | 261596 |
| **134** | NH_4_Cu_3_P_3_O_10_F_4_H_2_ | *C*2*/c* | 15 | CS | 0D | 884298^#^ |
| **135** | (NH_4_)_2_Co_3_(PO_3_F)_4_ | *P*2_1_*/c* | 14 | CS | 0D | 1427554^#^ |
| **136** | LiCo_2_P_4_O_10_F_4_ | *C*2*/c* | 15 | CS | 0D | 1427556^#^ |
| **137** | RbCo_3_P_3_O_10_F_4_ | *C*2*/c* | 15 | CS | 0D | 1427558^#^ |
| **138** | K_2_Co_3_(PO_3_F)_4_ | *P*2_1_*/c* | 14 | CS | 0D | 1427551^#^ |
| **139** | Cs_2_Mn_2_F_4_(PO_3_F)_2_ | *P*2_1_ | 4 | NCS | 0D | 424938 |
| **140** | KMnF_2_(PO_3_F) | *P*2_1_*/n* | 14 | CS | 0D | 424940 |
| **141** | Li_3_Mn(PO_3_F)_2_F_2_ | *P*2_1_*/c* | 14 | CS | 0D | 424941 |
| **142** | Na_2_Mn(HPO_3_F)F_3_ | *P*2_1_*/m* | 11 | CS | 0D | 424942 |
| **143** | Na_2_MnF_2_(PO_3_(O_0_._5_F_0_._5_))_2_ | *P*1 | 1 | NCS | 0D | 424943 |
| **144** | (NH_4_)Mn(PO_3_F)F_2_ | *P*2_1_*/n* | 14 | CS | 0D | 424945 |
| **145** | Rb_3_Mn_3_(PO_4_)(PO_3_F)_2_F_5_ | *Cc* | 9 | NCS | 0D | 424947 |
| **146** | (NH_4_)_3_Fe(PO_3_F)_2_F_2_ | *P*2_1_*/m* | 11 | CS | 0D | 425616 |
| **147** | Cs_2_Fe_2_F_3_(PO_3_F)_2_(PO_2_F_2_) | *Aba*2 | 41 | NCS | 0D | 425620 |
| **148** | KFe_2_(PO_2_F_2_)(PO_3_F)_2_F_2_ | *P*$\overline{\text{1}}$ | 2 | CS | 0D | 425625 |
| **149** | Fe_2_(PO_3_F)_3_ | *P*6_3_*/m* | 176 | CS | 0D | 425626 |
| **150** | NaFe(PO_3_F)_2_ | *P*2_1_*/c* | 14 | CS | 0D | 425628 |
| **151** | (NH_4_)_2_Fe_2_(PO_3_F)_2_FCl_2_ | *Pca*2_1_ | 29 | NCS | 0D | 425627 |
| **152** | Ba(PO_3_F) | *P*2_1_*/c* | 14 | CS | 0D | 425691 |
| **153** | Mn(PO_3_F)(H_2_O)_2_ | *P*$\overline{\text{1}}$ | 2 | CS | 0D | 428896 |
| **154** | Ag(PO_2_F_2_) | *C*2*/c* | 15 | CS | 0D | 429712 |
| **155** | Ag_9_(PO_2_F_2_)_14_ | *P*2_1_*/c* | 14 | CS | 0D | 429713 |
| **156** | Sr(PO_3_F) | *P*2_1_*/c* | 14 | CS | 0D | 429338 |
| **157** | Sr(PO_3_F)(H_2_O) | *P*2_1_*/c* | 14 | CS | 0D | 429339 |
| **158** | LiPO_2_F_2_ | *C*2*/c* | 15 | CS | 0D | 7122837^*^ |
| **159** | KFe(PO_3_F)F_2_ | *P*2_1_*/c* | 14 | CS | 0D | 11443 |
| **160** | K_2_Mn_3_(HPO_4_)_2_(PO_3_F)F_2_ | *P*2_1_*/c* | 14 | CS | 0D | 14932 |
| **161** | (NH_4_)Mn_3_(H_2_PO_4_)(PO_3_F)_2_F_2_ | *C*2*/c* | 15 | CS | 0D | 14946 |
| **162** | (NH_4_)_2_Mn_3_(HPO_4_)_2_(PO_3_F)F_2_ | *P*2_1_*/c* | 14 | CS | 0D | 14948 |
| **163** | Ba_2_Mn_2_(PO_3_F)F_6_ | *P*2_1_*/c* | 14 | CS | 0D | 16878 |
| **164** | Ba_2_Cu_2_(PO_3_F)F_6_ | *P*2_1_*/c* | 14 | CS | 0D | 16911 |
| **165** | Ba_2_Ni_2_(PO_3_F)F_6_ | *P*2_1_*/n* | 14 | CS | 0D | 16914 |
| **166** | Ba_2_Co_2_(PO_3_F)F_6_ | *P*2_1_*/c* | 14 | CS | 0D | 16920 |
| **167** | (NH_4_)_2_(PO_3_F) | *P*2_1_*/n* | 14 | CS | 0D | 17924 |
| **168** | K_0_._026_(NH_4_)_1_._974_(PO_3_F) | *Pna*2_1_ | 33 | NCS | 0D | 17925 |
| **169** | K_0_._02_(NH_4_)_1_._98_(PO_3_F) | *Pna*2_1_ | 33 | NCS | 0D | 17928 |
| **170** | K_0_._03_(NH_4_)_1_._97_(PO_3_F) | *Pna*2_1_ | 33 | NCS | 0D | 17929 |
| **171** | K_0_._03_(NH_4_)_1_._97_(PO_3_F) | *P*2_1_*/n* | 14 | CS | 0D | 17930 |
| **172** | K_0_._036_(NH_4_)_1_._964_(PO_3_F) | *P*2_1_*/n* | 14 | CS | 0D | 17931 |
| **173** | K_0_._03_(NH_4_)_1_._97_(PO_3_F) | *P*2_1_*/n* | 14 | CS | 0D | 17932 |
| **174** | K_0_._028_(NH_4_)_1_._972_(PO_3_F) | *P*2_1_*/n* | 14 | CS | 0D | 17933 |
| **175** | K_0_._0375_(NH_4_)_1_._9625_(PO_3_F) | *Pna*2_1_ | 33 | NCS | 0D | 17934 |
| **176** | K_0_._032_(NH_4_)_1_._968_(PO_3_F) | *Pna*2_1_ | 33 | NCS | 0D | 17935 |
| **177** | K_0_._034_(NH_4_)_1_._966_(PO_3_F) | *Pna*2_1_ | 33 | NCS | 0D | 17936 |
| **178** | K_0_._012_(NH_4_)_1_._988_(PO_3_F) | *P*2_1_*/n* | 14 | CS | 0D | 17937 |
| **179** | K_0_._039_(NH_4_)_1_._961_(PO_3_F) | *P*2_1_*/n* | 14 | CS | 0D | 17938 |
| **180** | K_0_._034_(NH_4_)_1_._966_(PO_3_F) | *Pna*2_1_ | 33 | NCS | 0D | 17939 |
| **181** | K_0_._034_(NH_4_)_1_._966_(PO_3_F) | *P*2_1_*/n* | 14 | CS | 0D | 17940 |
| **182** | K_0_._05_(NH_4_)_1_._95_(PO_3_F) | *P*2_1_*/n* | 14 | CS | 0D | 17941 |
| **183** | K_0_._04_(NH_4_)_1_._96_(PO_3_F) | *P*2_1_*/n* | 14 | CS | 0D | 17942 |
| **184** | K_0_._28_(NH_4_)_1_._72_(PO_3_F) | *Pna*2_1_ | 33 | NCS | 0D | 17943 |
| **185** | K_0_._28_(NH_4_)_1_._72_(PO_3_F) | *Pna*2_1_ | 33 | NCS | 0D | 17944 |
| **186** | K_0_._039_(NH_4_)_1_._961_(PO_3_F) | *P*2_1_*/n* | 14 | CS | 0D | 17945 |
| **187** | K_0_._291_(NH_4_)_1_._709_(PO_3_F) | *Pna*2_1_ | 33 | NCS | 0D | 17946 |
| **188** | K_0_._266_(N H_4_)_1_._734_(PO_3_F) | *P*2_1_*/n* | 14 | CS | 0D | 17947 |
| **189** | K_0_._28_(N H_4_)_1_._72_(PO_3_F) | *Pna*2_1_ | 33 | NCS | 0D | 17948 |
| **190** | K_0_._284_(N H_4_)_1_._716_(PO_3_F) | *Pna*2_1_ | 33 | NCS | 0D | 17949 |
| **191** | K_0_._272_(NH_4_)_1_._728_(PO_3_F) | *P*2_1_*/n* | 14 | CS | 0D | 17950 |
| **192** | K_0_._433_(N H_4_)_1_._567_(PO_3_F) | *Pna*2_1_ | 33 | NCS | 0D | 17951 |
| **193** | K_0_._263_(NH_4_)_1_._737_(PO_3_F) | *Pna*2_1_ | 33 | NCS | 0D | 17952 |
| **194** | K_0_._267_(N H_4_)_1_._733_(PO_3_F) | *P*2_1_*/n* | 14 | CS | 0D | 17953 |
| **195** | KLa(PO_2_F_2_)_4_ | *P*2_1_*/c* | 14 | CS | 0D | 18569 |
| **196** | (NH_4_)La(PO_2_F_2_)_4_ | *P*2_1_*/c* | 14 | CS | 0D | 18570 |
| **197** | NaFe(SO_4_)_1_._454_(PO_3_F)_0_._546_ | *C*2*/m* | 15 | CS | 0D | 105607 |
| **198** | (NH_4_)Mn_3_(PO_3_F)_2_(PO_2_F_2_)F_2_ | *C*2*/c* | 15 | CS | 0D | 141125 |
| **199** | (NH_4_)Co_3_(PO_3_F)_2_(PO_2_F_2_)F_2_ | *C*2*/c* | 15 | CS | 0D | 141126 |
| **200** | (NH_4_)_2_Ba(PO_2_F_2_)_4_ | *P*2_1_*/n* | 14 | CS | 0D | 33092 |
| **201** | Ba(PO_2_F_2_)_2_ | *I*$\overline{\text{4}}$2*d* | 122 | NCS | 0D | 33093 |
| **202** | (NH_4_)Mg(PO_2_F_2_)_3_ | *CmCm* | 63 | CS | 0D | 33094 |
| **203** | (NH_4_)Sr(PO_2_F_2_)_3_ | *P*$\overline{\text{1}}$ | 2 | CS | 0D | 33095 |
| **204** | (NH_4_)_2_Fe_2_F_2_(PO_4_)(PO_3_F)OH | *P*2_1_*/n* | 14 | CS | 0D | 136727 |
| **205** | (NH_4_)_2_V_2_F_2_(PO_4_)(PO_3_F)OH | *P*2_1_*/n* | 14 | CS | 0D | 136728 |
| **206** | (NH_4_)_2_Co(PO_3_F)_2_(H_2_O)_2_ | *C*2*/m* | 15 | CS | 0D | 143455 |
| **207** | Cd(PO_3_F)(H_2_O)_2_ | *P*$\overline{\text{1}}$ | 2 | CS | 0D | 143456 |
| **208** | (NH_4_)_2_Mg(PO_3_F)_2_(H_2_O)_2_ | *C*2*/m* | 15 | CS | 0D | 143457 |
| **209** | (NH_4_)_2_Zn_3_(PO_3_F)_4_(H_2_O) | *I*$\overline{\text{4}}$3*d* | 220 | NCS | 0D | 143458 |
| **210** | (NH_4_)_2_Mn(PO_3_F)_2_(H_2_O)_2_ | *P*2_1_*/n* | 14 | CS | 0D | 143459 |
| **211** | (NH_4_)Cu_2_(H_3_O_2_)(PO_3_F)_2_ | *C*2*/m* | 15 | CS | 0D | 143460 |
| **212** | (NH_4_)Cr(PO_3_F)_2_(H_2_O)_6_ | *R*$\overline{\text{3}}$*m* | 166 | CS | 0D | 143461 |
| **213** | Zn(PO_3_F)(H_2_O)_2_._5_ | *P*$\overline{\text{3}}$ | 2 | CS | 0D | 143464 |
| **214** | (NH_4_)_2_Ni(PO_3_F)_2_(H_2_O)_6_ | *P*2_1_*/c* | 14 | CS | 0D | 143465 |
| **215** | Pb_2_(PO_3_F)Cl_2_(H_2_O) | *Pnma* | 62 | CS | 0D | 143462 |
| **216** | (NH_4_)_2_Zn(PO_3_F)_2_(H_2_O)_0_._2_ | *C*2*/c* | 15 | CS | 0D | 143463 |
| **217** | Cr_2_(PO_3_F)_3_(H_2_O)_18_._8_ | *P*$\overline{\text{1}}$ | 2 | CS | 0D | 143466 |
| **218** | NaPO_2_F_2_ | *P*$\overline{\text{1}}$ | 2 | CS | 0D | 143623 |
| **219** | Na_1_._5_Rb_0_._5_PO_3_F·H_2_O | *Pmn*2_1_ | 31 | NCS | 0D | 2045145^#^ |
| **220** | K_3_Sc_3_(PO_4_)(PO_3_F)_2_F_5_ | *Cc* | 9 | NCS | 0D | / |
| **221** | Cd_2_._5_(NH_4_)_2_(PO_3_F)_3_Cl·2H_2_O | *P*2_1_*/c* | 14 | CS | 0D | 2117714^#^ |
| **Fluorosulfates** | | | | | | |
| **222** | KSO_3_F | *Pnma* | 62 | CS | 0D | 16864 |
| **223** | NH_4_SO_3_F | *Pnma* | 62 | CS | 0D | 16146 |
| **224** | XeF(SO_3_F) | *Pbca* | 61 | CS | 0D | 10357 |
| **225** | Li(SO_3_F) | *C*2*/m* | 15 | CS | 0D | 1384 |
| **226** | I_2_(SO_3_F)_2_ | *P*2_1_2_1_2_1_ | 19 | NCS | 0D | 49562 |
| **227** | Se_10_(SO_3_F)_2_ | *P*2_1_2_1_2_1_ | 19 | NCS | 0D | 61071 |
| **228** | Sn(SO_3_F)_2_ | *P*2_1_*/c* | 14 | CS | 0D | 66483 |
| **229** | Au_2_(SO_3_F)_6_ | *P*2_1_*/c* | 14 | CS | 0D | 69984 |
| **230** | Cs(SO_3_F) | *I*4_1_*/amd* | 141 | CS | 0D | 82520 |
| **231** | Cs(Sb(SO_3_F)_6_) | *R*$\overline{\text{3}}$ | 148 | CS | 0D | 82524 |
| **232** | Cs(SO_3_F) | *P*2_1_*/c* | 14 | CS | 0D | 82520 |
| **233** | CsH(SO_3_F)_2_ | *C*2*/c* | 15 | CS | 0D | 82521 |
| **234** | Cs(Au(SO_3_F)_4_) | *C*2*/c* | 15 | CS | 0D | 82522 |
| **235** | Cs_2_(Pt(SO_3_F)_6_) | *P*321 | 150 | NCS | 0D | 82523 |
| **236** | Cs(Sb(SO_3_F)_6_) | *R*$\overline{\text{3}}$ | 148 | CS | 0D | 82524 |
| **237** | (TcO_3_)(SO_3_F) | *P*2_1_*/c* | 14 | CS | 0D | 418710 |
| **238** | Ag(SO_3_F)_2_ | *P*2_1_*/c* | 14 | CS | 0D | 422414 |
| **239** | Ag(SO_3_F) | *P*2_1_*/m* | 11 | CS | 0D | 183644 |
| **240** | Ag_3_(SO_3_F)_4_ | *P*2_1_*/c* | 14 | CS | 0D | 422417 |
| **241** | Na(SO_3_F) | *P*6_3_*/mmc* | 194 | CS | 0D | 424112 |
| **242** | Rb(SO_3_F) | *Pnma* | 62 | CS | 0D | 424113 |
| **243** | Tl(SO_3_F) | *Pnma* | 62 | CS | 0D | 424114 |
| **244** | Xe(SO_3_F)_2_ | *P*2_1_*/n* | 14 | CS | 0D | 195088 |
| **245** | K_2_(BeS)O_4_F_2_ | *P*2_1_2_1_2_1_ | 19 | NCS | 0D | 2203573^#^ |
| **Fluorosulfite** | | | | | | |
| **246** | KSO_2_F | *P*2_1_*/m* | 11 | CS | 0D | 89602 |
| **247** | RbSO_2_F | *P*2_1_*/m* | 11 | CS | 0D | 89603 |
| **248** | α-CsSO_2_F | *Pnma* | 62 | CS | 0D | 93070 |
| **249** | β-CsSO_2_F | *R*3*m* | 160 | NCS | 0D | 93080 |
| **Fluoroiodate** | | | | | | |
| **250** | KIO_2_F_2_ | *Pca*2_1_ | 29 | NCS | 0D | 26600 |
| **251** | CsIOF_4_ | *Pnma* | 62 | CS | 0D | 2754 |
| **252** | SbF_5_(IO_2_F_3_) | *P*2_1_*/c* | 14 | CS | 0D | 201199 |
| **253** | (IOF_2_)(IO_2_F_4_) | *P*2_1_*/c* | 14 | CS | 0D | 201202 |
| **254** | Na(IO_2_F_2_) | *Cmcm* | 63 | CS | 0D | 260003 |
| **255** | RbIO_2_F_2_ | *Pca*2_1_ | 29 | NCS | 0D | 291600 |
| **256** | Cs_3_(IO_2_F_2_)_3_(H_2_O) | *Pnma* | 62 | CS | 0D | 255454 |
| **257** | CsIO_2_F_2_ | *Pca*2_1_ | 29 | NCS | 0D | 255455 |
| **258** | Cs(IO_2_F_2_)_2_(H_5_O_2_) | *P*2_1_*/c* | 14 | CS | 0D | 255456 |
| **259** | SrI_2_O_5_F_2_ | *P*2_1_*/c* | 14 | CS | 0D | 132693 |
| **260** | [GaF(H_2_O)][IO_3_F] | *Pca*2_1_ | 29 | NCS | 0D | 2083798^#^ |
| **261** | Ba(IO_2_F_2_)_2_ | *P*2_1_*/c* | 14 | CS | 0D | 132695 |
| **262** | Cs_2_MoO_2_F_3_(IO_2_F_2_) | *Cmc*2_1_ | 36 | NCS | 0D | 113179 |
| **263** | Rb_2_MoO_2_F_3_(IO_2_F_2_) | *Cmc*2_1_ | 36 | NCS | 0D | 113180 |
| **264** | NaGa(IO_3_F)(IO_3_)_2_ | *P*2_1_*/c* | 14 | CS | 0D | 2091131^#^ |
| **265** | BaI_2_O_5_F_2_ | *P*2_1_*/c* | 14 | CS | 0D | 2064589^#^ |
| **266** | BaIO_2_F_3_ | *Cmca* | 64 | CS | 0D | 2064590^#^ |
| **267** | NH_4_IO_2_F_2_ | *Pca*2_1_ | 29 | NCS | 0D | 2156529^#^ |
| **268** | (NH_4_)_3_(IO_2_F_2_)_3_·H_2_O | *Pnma* | 62 | CS | 0D | 2156530^#^ |
| **269** | Cs_2_VOF_4_(IO_2_F_2_) | *Cmc*2_1_ | 36 | NCS | 0D | 2168878^#^ |
| **Fluoroarsenate** | | | | | | |
| **270** | KSeO_2_F | *P*2_1_*/m* | 11 | CS | 0D | 78398 |
| **271** | RbSeO_2_F | *Pm*$\overline{\text{3}}$*m* | 221 | CS | 0D | 78399 |
| **272** | CsSeO_2_F | *Pm*$\overline{\text{3}}$*m* | 221 | CS | 0D | 78400 |
| **Fluorotellurate** | | | | | | |
| **273** | KTeOF_3_ | *P*2_1_ | 4 | NCS | 0D | 201155 |
| **274** | N H4TeO(OH)F2 | *P*2_1_*/n* | 14 | CS | 0D | 201156 |
| **275** | Rb(Te(OH)F_4_) | *Cc* | 9 | NCS | 0D | 155197 |
| **276** | K(Te(OH)F_4_) | *P*2_1_ | 4 | NCS | 0D | 155199 |
| **277** | Rb_2_(Te_2_O(OH)F_7_) | *P*2_1_ | 4 | NCS | 0D | 155201 |
| **278** | (NH_4_)_2_(Te_2_O_2_F_6_) | *P*4_2_*/n* | 86 | CS | 0D | 155202 |
| **279** | KTeO_2_F | *P*2_1_*/n* | 14 | CS | 0D | 411068 |
| **280** | Cs2(TeOF4) | *Cmcm* | 63 | CS | 0D | 411626 |
| **281** | KTeOF_3_ | *P*4_2_*/n* | 86 | CS | 0D | 411806 |
| **282** | K_2_(TeOF_4_) | *Cmcm* | 63 | CS | 0D | 412955 |
| **283** | Hg(TeO_2_F(OH)) | *Pca*2_1_ | 29 | NCS | 0D | 115597 |
| **284** | In_3_TeO_3_F_7_ | *Cmmm* | 65 | CS | 0D | 180142 |
| **285** | Pb_2_Al_3_F_3_(Te_6_F_2_O_16_) | *P*4*/mbm* | 127 | CS | 0D | 120978 |
| **286** | Ba_2_Ga_3_F_3_(Te_6_F_2_O_16_) | *P*4*/mbm* | 127 | CS | 0D | 120979 |
| **287** | Pb_2_Ga_3_F_3_(Te_6_F_2_O_16_) | *P*4*/mbm* | 127 | CS | 0D | 120980 |
| **288** | BaF_2_TeF_2_(OH)_2_ | *Pmn*2_1_ | 31 | NCS | 0D | 17830 |
| **Fluoroselenite** | | | | | | |
| **289** | Cs_2_(As_2_F_10_O) | *P*2_1_*/m* | 11 | CS | 0D | 21061 |
| **290** | K_2_As_2_F_8_O_2_ | *P*2_1_*/n* | 14 | CS | 0D | 9027 |
| **291** | Rb_2_As_2_F_8_O_2_ | *P*2_1_*/n* | 14 | CS | 0D | 9028 |
| **292** | Cs_2_As_2_F_8_O_2_ | *P*2_1_*/c* | 14 | CS | 0D | 6070 |
| **293** | Rb_2_As_2_F_10_OH_2_O | *Pnma* | 62 | CS | 0D | 2123 |
| **294** | K_2_As_2_F_10_OH_2_O | *Pnma* | 62 | CS | 0D | 2124 |
| **295** | K(AsF_5_(OH)) | *Pbcn* | 60 | CS | 0D | 59414 |
| **296** | K(AsF_4_(OH)_2_) | *C*2*/c* | 15 | CS | 0D | 15625 |
| **297** | GaF(AsO_2_F(OH))_2_ | *I*4*/mcm* | 140 | CS | 0D | 429382 |
| **Fluoroborophosphates** | | | | | | |
| **298** | NH_4_(BPO_4_F) | *P*2_1_3 | 198 | NCS | 3D | 170949 |
| **299** | Na_3_B_2_PO_5_F_4_-I | *P*2_1_*/n* | 14 | CS | 0D | 425964 |
| **300** | Na_3_B_2_PO_5_F_4_-II | *Cmcm* | 63 | CS | 0D | 425965 |
| **301** | KB(PO_4_)F | *Cc* | 9 | NCS | 2D | 431892 |
| **302** | RbB(PO_4_)F | *P*2_1_3 | 198 | NCS | 3D | 13417 |
| **303** | CsB(PO_4_)F | *P*2_1_3 | 198 | NCS | 3D | 13418 |
| **304** | K_3_B_2_PO_5_F_4_ | *P*2_1_*/n* | 14 | CS | 0D | 403275 |
| **305** | Cs_3_B_2_PO_5_F_4_ | *P*2_1_*/n* | 14 | CS | 0D | 403274 |
| **306** | (NH_4_)_2_BPO_4_F_2_ | *P*2_1_ | 4 | NCS | 1D | 1884383^#^ |
| **307** | (NH_4_)_3_B_11_PO_19_F_3_ | *R*3 | 146 | NCS | 2D | 2153289^#^ |
| **308** | Rb_3_B_2_PO_5_F_4_ | *P*2_1_*/c* | 14 | CS | 0D | 2129537^#^ |
| **309** | K_4_B_2_P_2_O_9_F_2_ | *P*2_1_*/c* | 14 | CS | 0D | 2129536^#^ |
| **Fluorophosphorborates** | | | | | | |
| **310** | Li(B(PO_2_F_2_)_4_) | *P*2_1_*/c* | 14 | CS | 0D | 195129 |
| **Fluoroborate-fluorophosphate** | | | | | | |
| **311** | (NH_4_)_3_[PO_3_F][BF_4_] | *P*2_1_*/m* | 11 | CS | 0D | 2094843^#^ |
| **Fluoroboroarsenate** | | | | | | |
| **312** | (NH_4_)(BAsO_4_F) | *Cc* | 198 | NCS | 2D | 420517 |
| **Sulfate-fluorophosphates** | | | | | | |
| **313** | K_4_(PO_2_F_2_)_2_(S_2_O_7_) | *C*2*/c* | 15 | CS | 0D | 22737 |
| **Fluorooxosilicophosphate** | | | | | | |
| **314** | K_4_Si_3_P_2_O_7_F_12_ | *Pnma* | 62 | CS | 1D | 256232 |
| **315** | CsSiP_2_O_7_F | *P*2_1_ | 4 | NCS | 2D | 17974 |
| **316** | Na_4_Si_2_PO_4_F_9_ | *P*$\overline{\text{1}}$ | 2 | CS | 1D | 1952892^#^ |
| **Sulfate-fluorotellurates** | | | | | | |
| **317** | Na_2_Te(OH)F_3_(SO_4_) | *P* 2_1_*/c* | 14 | CS | 0D | 20729 |
| **318** | (NH_4_)_2_Te(OH)F_3_(SO_4_) | *P*1 | 1 | NCS | 0D | 20730 |
| **Fluoroarsenate-fluorosulfate** | | | | | | |
| **319** | ((XeF)_2_SO_3_F)(AsF_6_) | *P*2_1_*/n* | 14 | CS | 0D | 200036 |
| **320** | Hg(AsF_6_)(SO_3_F) | *P*2_1_*/c* | 14 | CS | 0D | 429286 |

The code with “*” means COD code. The code with “#” means CCDC code.

**Table S2.** Summary of selected fluorooxysalts with experimental SHG response.

| **No.** | **Formula** | **SHG response (×KDP)** |
| --- | --- | --- |
| **1** | Na_2_B_6_O_9_F_2_ | 0.08 |
| **2** | RbB(PO_4_)F | 0.4 |
| **3** | K_10_B_13_O_15_F_19_ | 0.4 |
| **4** | Rb_10_B_13_O_15_F_19_ | 0.5 |
| **5** | Na_1_._5_Rb_0_._5_PO_3_F·H_2_O | 0.55 |
| **6** | CsB(PO_4_)F | 0.6 |
| **7** | CsKB_8_O_12_F_2_·CsI | 0.6 |
| **8** | CsSiP_2_O_7_F | 0.7 |
| **9** | Na_4_B_8_O_9_F_10_ | 0.7 |
| **10** | (NH_4_)_2_BPO_4_F_2_ | 0.8 |
| **11** | RbB_4_O_6_F | 0.8 |
| **12** | K_3_Sc_3_(PO_4_)(PO_3_F)_2_F_5_ | 0.9 |
| **13** | Li_2_B_6_O_9_F_2_ | 0.9 |
| **14** | NaB_4_O_6_F | 0.9 |
| **15** | KB(PO_4_)F | 1 |
| **16** | (NH_4_)_2_(PO_3_F) | 1 |
| **17** | CsRbB_8_O_12_F_2_ | 1.1 |
| **18** | (NH_4_)Na(PO_3_F)(H_2_O) | 1.1 |
| **19** | Hg(TeO_2_F(OH)) | 1.1 |
| **20** | (NH_4_)_3_B_11_PO_19_F_3_ | 1.2 |
| **21** | NH_4_IO_2_F_2_ | 1.2 |
| **22** | LiB_6_O_9_F | 1.4 |
| **23** | SrB_5_O_7_F_3_ | 1.6 |
| **24** | CsKB_8_O_12_F_2_ | 1.9 |
| **25** | CsB_4_O_6_F | 1.9 |
| **26** | CaB_5_O_7_F_3_ | 2 |
| **27** | (NH_4_)(BAsO_4_F) | 2 |
| **28** | MgB_5_O_7_F_3_ | 2.4 |
| **29** | CsIO_2_F_2_ | 3 |
| **30** | BaF_2_TeF_2_(OH)_2_ | 3 |
| **31** | NH_4_B_4_O_6_F | 3 |
| **32** | SnB_2_O_3_F_2_ | 4 |
| **33** | RbIO_2_F_2_ | 4 |
| **34** | Cs_2_MoO_2_F_3_(IO_2_F_2_) | 4.5 |
| **35** | Rb_2_MoO_2_F_3_(IO_2_F_2_) | 5 |
| **36** | Cs_2_VOF_4_(IO_2_F_2_) | 5 |
| **37** | PbB_5_O_7_F_3_ | 6 |
| **38** | [GaF(H_2_O)][IO_3_F] | 10 |
| **39** | BiB_2_O_4_F | 12.1 |
| **40** | PbB_2_O_3_F_2_ | 13 |

**Table S3.** Summary of selected fluorooxysalts with calculated or experimental birefringence.

| **No.** | **Formula** | **Birefringence** |
| --- | --- | --- |
| **1** | Cs_3_B_3_O_3_F_6_ | 0.0069@1064 nm cal. |
| **2** | CsSiP_2_O_7_F | 0.009@1064 nm cal. |
| **3** | Hg(TeO_2_F(OH)) | 0.009@1064 nm cal. |
| **4** | Ba(PO_3_F) | 0.01@1064 nm cal. |
| **5** | (NH_4_)_3_[PO_3_F][BF_4_] | 0.012@1064 nm cal. |
| **6** | K_4_(PO_2_F_2_)_2_(S_2_O_7_) | 0.015@1064 nm cal. |
| **7** | Cs_2_PO_3_F | 0.018@1064 nm cal. |
| **8** | BiB_2_O_4_F | 0.018@1064 nm cal. |
| **9** | Rb_2_PO_3_F | 0.02@1064 nm cal. |
| **10** | K_2_(PO_3_F) | 0.021@1064 nm cal. |
| **11** | NaK_3_(PO_3_F)_2_ | 0.021@1064 nm cal. |
| **12** | Rb(SO_3_F) | 0.022@1064 nm cal. |
| **13** | K_2_(P_2_O_5_F_2_) | 0.023@1064 nm cal. |
| **14** | NH_4_IO_2_F_2_ | 0.023@1064 nm cal. |
| **15** | K_3_Sc_3_(PO_4_)(PO_3_F)_2_F_5_ | 0.024@1064 nm cal. |
| **16** | (NH_4_)_3_(IO_2_F_2_)_3_·H_2_O | 0.024@1064 nm cal. |
| **17** | Rb_10_B_13_O_15_F_19_ | 0.026@1064 nm cal. |
| **18** | (NH_4_)_2_(PO_3_F) | 0.027@1064 nm cal. |
| **19** | Na_2_(PO_3_F) | 0.028@1064 nm cal. |
| **20** | (NH_4_)(BAsO_4_F) | 0.03@1064 nm cal. |
| **21** | K_10_B_13_O_15_F_19_ | 0.03@1064 nm cal. |
| **22** | K_2_B_3_O_4_F_3_ | 0.031@1064 nm cal. |
| **23** | Cs(SO_3_F) | 0.031@1064 nm cal. |
| **24** | KCsB_3_O_4_F_3_ | 0.032@1064 nm cal. |
| **25** | Sr(PO_3_F) | 0.034@1064 nm cal. |
| **26** | Cd_2_._5_(NH_4_)_2_(PO_3_F)_3_Cl·2H_2_O | 0.035@1064 nm cal. |
| **27** | Na_4_B_8_O_9_F_10_ | 0.036@1064 nm cal. |
| **28** | KNaB_3_O_4_F_3_ | 0.04@1064 nm cal. |
| **29** | NaRbB_3_O_4_F_3_ | 0.04@1064 nm cal. |
| **30** | KB(PO_4_)F | 0.044@1064 nm cal. |
| **31** | NH_4_SO_3_F | 0.044@1064 nm cal. |
| **32** | α-BaB_4_O_5_F_4_ | 0.044@1064 nm cal. |
| **33** | Cs(PO_2_F_2_) | 0.045@1064 nm cal. |
| **34** | (NH_4_)(PO_2_F_2_) | 0.046@1064 nm cal. |
| **35** | CsIO_2_F_2_ | 0.046@1064 nm cal. |
| **36** | K(PO_2_F_2_) | 0.047@1064 nm cal. |
| **37** | β-BaB_4_O_5_F_4_ | 0.047@1064 nm cal. |
| **38** | Li_2_B_3_O_4_F_3_ | 0.05@1064 nm cal. |
| **39** | NH_4_B_6_O_9_F_2_ | 0.05@1064 nm cal. |
| **40** | K_2_B_6_O_9_F_2_ | 0.053@1064 nm cal. |
| **41** | (NH_4_)Na(PO_3_F)(H_2_O) | 0.053@589.3 nm exp. |
| **42** | Ba_3_B_10_O_17_F_2_·0.1KF | 0.054@1064 nm cal. |
| **43** | BaB_5_O_8_F·xH_2_O | 0.055@1064 nm cal. |
| **44** | Li(SO_3_F) | 0.057@1064 nm cal. |
| **45** | LiB_6_O_9_F | 0.06@1064 nm cal. |
| **46** | SrB_5_O_7_F_3_ | 0.07@1064 nm cal. |
| **47** | Li_2_B_6_O_9_F_2_ | 0.07@1064 nm cal. |
| **48** | MgB_5_O_7_F_3_ | 0.07@1064 nm cal. |
| **49** | CaB_5_O_7_F_3_ | 0.07@1064 nm cal. |
| **50** | PbB_5_O_8_F | 0.073@1064 nm cal. |
| **51** | Sn(PO_3_F) | 0.075@1064 nm cal. |
| **52** | BaF_2_TeF_2_(OH)_2_ | 0.078@589 nm exp. |
| **53** | CsKB_8_O_12_F_2_·CsI | 0.08@1064 nm cal. |
| **54** | Na_2_B_6_O_9_F_2_ | 0.08@589.3 nm exp. |
| **55** | Na_3_B_7_O_11_F_2_ | 0.083@193 nm cal. |
| **56** | BaB_4_O_6_F_2_ | 0.085@1064 nm cal. |
| **57** | Ba(B_2_OF_3_(OH)_2_)_2_ | 0.086@1064 nm cal. |
| **58** | Cs(IO_2_F_2_)_2_(H_5_O_2_) | 0.086@1064 nm cal. |
| **59** | Cs_2_VOF_4_(IO_2_F_2_) | 0.088@1064 nm cal. |
| **60** | (NH_4_)_3_B_11_PO_19_F_3_ | 0.088@1064 nm cal. |
| **61** | SrB_4_O_6_F_2_ | 0.089@1064 nm cal. |
| **62** | RbB_3_O_4_F_2_ | 0.09@1064 nm cal. |
| **63** | CaB_4_O_6_F_2_ | 0.091@1064 nm cal. |
| **64** | Ba(IO_2_F_2_)_2_ | 0.092@1064 nm cal. |
| **65** | Cs_3_(IO_2_F_2_)_3_(H_2_O) | 0.093@1064 nm cal. |
| **66** | NaB_3_O_4_F(OH) | 0.097@1064 nm cal. |
| **67** | RbB_4_O_6_F | 0.102@1064 nm cal. |
| **68** | CsKB_8_O_12_F_2_ | 0.105@1064 nm cal. |
| **69** | CsB_4_O_6_F | 0.114@1064 nm exp. |
| **70** | BaB_8_O_12_F_2_ | 0.116@1064 nm cal. |
| **71** | NH_4_B_4_O_6_F | 0.117@1064 nm exp. |
| **72** | NaB_4_O_6_F | 0.12@1064 nm cal. |
| **73** | (NH_4_)_4_[B_12_O_16_F_4_(OH)_4_] | 0.12@589.3 nm exp. |
| **74** | PbB_5_O_7_F_3_ | 0.12@1064 nm cal. |
| **75** | LiB_5_O_5_F_2_(OH)_4_ | 0.124@546 nm cal. |
| **76** | Na(B_3_O_3_F_2_(OH)_2_)(B(OH)_3_) | 0.125@546 nm cal. |
| **77** | BaIO_2_F_3_ | 0.133@1064 nm cal. |
| **78** | [GaF(H_2_O)][IO_3_F] | 0.142@1064 nm cal. |
| **79** | BaI_2_O_5_F_2_ | 0.174@1064 nm cal. |
| **80** | SrI_2_O_5_F_2_ | 0.18@1064 nm cal. |
| **81** | NaGa(IO_3_F)(IO_3_)_2_ | 0.197@1064 nm cal. |
| **82** | Cs_2_MoO_2_F_3_(IO_2_F_2_) | 0.204@1064 nm cal. |
| **83** | Rb_2_MoO_2_F_3_(IO_2_F_2_) | 0.217@1064 nm cal. |

**Table S4.** Summary of selected fluorooxysalts with calculated or experimental band gap.

| **No.** | **Formula** | **Band gap (eV)** |
| --- | --- | --- |
| **1** | Hg(TeO_2_F(OH)) | 3.26 exp. |
| **2** | Cs_2_MoO_2_F_3_(IO_2_F_2_) | 3.43 exp. |
| **3** | Rb_2_MoO_2_F_3_(IO_2_F_2_) | 3.77 exp. |
| **4** | RbIO_2_F_2_ | 4.20 exp. |
| **5** | NaGa(IO_3_F)(IO_3_)_2_ | 4.27 exp. |
| **6** | BaIO_2_F_3_ | 4.27 exp. |
| **7** | BaI_2_O_5_F_2_ | 4.30 exp. |
| **8** | [GaF(H_2_O)][IO_3_F] | 4.34 exp. |
| **9** | Cs(IO_2_F_2_)_2_(H_5_O_2_) | 4.40 exp. |
| **10** | BiB_2_O_4_F | 4.43 exp. |
| **11** | Cs_3_(IO_2_F_2_)_3_(H_2_O) | 4.50 exp. |
| **12** | CsIO_2_F_2_ | 4.50 exp. |
| **13** | NH_4_IO_2_F_2_ | 4.53 exp. |
| **14** | (NH_4_)_3_(IO_2_F_2_)_3_·H_2_O | 4.55 exp. |
| **15** | Cs_2_VOF_4_(IO_2_F_2_) | 4.82 exp. |
| **16** | Sn(PO_3_F) | 4.83 cal. |
| **17** | SrI_2_O_5_F_2_ | 4.96 exp. |
| **18** | SnB_2_O_3_F_2_ | 4.96 exp. |
| **19** | Sr(PO_3_F) | 5.36 cal. |
| **20** | Ba(IO_2_F_2_)_2_ | 5.39 exp. |
| **21** | K_3_B_3_O_3_F_6_ | 5.40 exp. |
| **22** | Rb_3_B_2_PO_5_F_4_ | 5.48 exp. |
| **23** | PbB_5_O_7_F_3_ | 5.51 exp. |
| **24** | K_4_B_2_P_2_O_9_F_2_ | 5.54 exp. |
| **25** | PbB_2_O_3_F_2_ | 5.64 exp. |
| **26** | CsKB_8_O_12_F_2_·CsI | 5.74 exp. |
| **27** | (NH_4_)(BAsO_4_F) | 5.90 exp. |
| **28** | BaF_2_TeF_2_(OH)_2_ | 5.90 exp. |
| **29** | Na_2_(PO_3_F) | 6.14 cal. |
| **30** | KB(PO_4_)F | 6.22 exp. |
| **31** | (NH_4_)_2_BPO_4_F_2_ | 6.22 exp. |
| **32** | CsB(PO_4_)F | 6.22 exp. |
| **33** | Cd_2_._5_(NH_4_)_2_(PO_3_F)_3_Cl·2H_2_O | 6.22 exp. |
| **34** | K_3_Sc_3_(PO_4_)(PO_3_F)_2_F_5_ | 6.22 exp. |
| **35** | Na_1_._5_Rb_0_._5_PO_3_F·H_2_O | 6.22 exp. |
| **36** | RbB(PO_4_)F | 6.22 exp. |
| **37** | K_4_Si_3_P_2_O_7_F_12_ | 6.34 cal. |
| **38** | Na_4_Si_2_PO_4_F_9_ | 6.45 cal. |
| **39** | KSO_3_F | 6.50 exp. |
| **40** | CsSiP_2_O_7_F | 6.52 exp. |
| **41** | PbB_5_O_8_F | 6.52 exp. |
| **42** | (NH_4_)_3_[PO_3_F][BF_4_] | 6.52 exp. |
| **43** | β-BaBOF_3_ | 6.53 exp. |
| **44** | α-BaBOF_3_ | 6.53 exp. |
| **45** | γ-BaBOF_3_ | 6.53 exp. |
| **46** | NaB_3_O_4_F(OH) | 6.56 exp. |
| **47** | Cs_4_B_4_O_3_F_10_ | 6.58 cal. |
| **48** | Ba_2_B_7_O_12_F | 6.67 exp. |
| **49** | K_2_(PO_3_F) | 6.69 cal. |
| **50** | NH_4_SO_3_F | 6.70 cal. |
| **51** | Cs(SO_3_F) | 6.75 cal. |
| **52** | Rb(SO_3_F) | 6.75 cal. |
| **53** | Rb_2_PO_3_F | 6.76 cal. |
| **54** | (NH_4_)_3_B_11_PO_19_F_3_ | 6.78 exp. |
| **55** | Li_2_(Na_0_._9_K_0_._1_)B_5_O_8_F_2_ | 6.88 exp. |
| **56** | BaB_8_O_12_F_2_ | 6.89 cal. |
| **57** | LiPO_2_F_2_ | 6.89 exp. |
| **58** | BaB_2_O_3_F_2_ | 6.89 cal. |
| **59** | NaK_3_(PO_3_F)_2_ | 6.91 cal. |
| **60** | Cs_2_PO_3_F | 6.94 cal. |
| **61** | K_3_B_6_O_9_F_3_ | 6.98 exp. |
| **62** | K_4_(PO_2_F_2_)_2_(S_2_O_7_) | 6.99 cal. |
| **63** | (NH_4_)_2_(PO_3_F) | 7.01 exp. |
| **64** | (NH_4_)Na(PO_3_F)(H_2_O) | 7.05 exp. |
| **65** | KCsB_3_O_4_F_3_ | 7.10 cal. |
| **66** | (NH_4_)_4_[B_12_O_16_F_4_(OH)_4_] | 7.10 cal. |
| **67** | KNaB_3_O_4_F_3_ | 7.12 cal. |
| **68** | NH_4_B_6_O_9_F_2_ | 7.16 cal. |
| **69** | Cs_3_B_3_O_3_F_6_ | 7.16 cal. |
| **70** | Rb_10_B_13_O_15_F_19_ | 7.32 cal. |
| **71** | Na_2_B_6_O_9_F_2_ | 7.34 exp. |
| **72** | K_2_B_3_O_4_F_3_ | 7.40 cal. |
| **73** | K_2_B_6_O_9_F_2_ | 7.41 cal. |
| **74** | NaB_4_O_6_F | 7.57 cal. |
| **75** | K_10_B_13_O_15_F_19_ | 7.64 cal. |
| **76** | RbB_3_O_4_F_2_ | 7.67 cal. |
| **77** | Li(SO_3_F) | 7.68 cal. |
| **78** | Na_3_B_7_O_11_F_2_ | 7.69 cal. |
| **79** | CaB_4_O_6_F_2_ | 7.72 cal. |
| **80** | RbB_4_O_6_F | 7.73 cal. |
| **81** | Na_4_B_8_O_9_F_10_ | 7.73 cal. |
| **82** | K_2_(P_2_O_5_F_2_) | 7.74 cal. |
| **83** | CsKB_8_O_12_F_2_ | 7.76 cal. |
| **84** | SrB_4_O_6_F_2_ | 7.81 cal. |
| **85** | MgB_5_O_7_F_3_ | 7.86 cal. |
| **86** | NH_4_B_4_O_6_F | 7.87 exp. |
| **87** | Ba(PO_3_F) | 7.90 cal. |
| **88** | Na(B_3_O_3_F_2_(OH)_2_)(B(OH)_3_) | 7.92 exp. |
| **89** | K(PO_2_F_2_) | 7.95 cal. |
| **90** | LiB_5_O_5_F_2_(OH)_4_ | 7.99 cal. |
| **91** | Cs(PO_2_F_2_) | 7.99 cal. |
| **92** | CsB_4_O_6_F | 8.00 exp. |
| **93** | Li_2_B_6_O_9_F_2_ | 8.05 cal. |
| **94** | KCs_2_B_3_O_3_F_6_ | 8.13 cal. |
| **95** | Rb_3_B_3_O_3_F_6_ | 8.15 cal. |
| **96** | (NH_4_)(PO_2_F_2_) | 8.15 cal. |
| **97** | β-BaB_4_O_5_F_4_ | 8.30cal. |
| **98** | Ba(B_2_OF_3_(OH)_2_)_2_ | 8.35 cal. |
| **99** | LiB_6_O_9_F | 8.37 cal. |
| **100** | α-BaB_4_O_5_F_4_ | 8.4 cal. |
| **101** | Li_2_B_3_O_4_F_3_ | 8.43 cal. |
| **102** | BaB_4_O_6_F_2_ | 8.44 cal. |
| **103** | SrB_5_O_7_F_3_ | 8.58 cal. |
| **104** | CaB_5_O_7_F_3_ | 8.75 cal. |
